# Supplementary material for: Colchicine combination therapy increases treatment tolerance in patients with arthritis: A systematic review and meta-analysis
Source: PLoS One. 2024 Dec 30;19(12):e0316126. doi: 10.1371/journal.pone.0316126 (PMC11684588; doi:10.1371/journal.pone.0316126)
Supplement: S1 File — (DOCX) [file pone.0316126.s001.docx]

Efficacy and safety of colchicine in combination therapy for arthritis: a systematic review and meta-analysis

Changwei Zhao^1，2^, Xiaogang Hao^2^, Wenjun Cai^3^, Ling-Feng Zeng^4^，Wenhai Zhao^2^ ^*^，Xiangxin Li^2^ ^*^

1 Changchun University of Chinese Medicine, Changchun, China;

2 The Affiliated Hospital of Changchun University of Chinese Medicine, Changchun,

China;

3 Third Affiliated Clinical Hospital to Changchun University of Chinese Medicine， Changchun, China;

4 The Second Affiliated Hospital of Guangzhou University of Chinese Medicine, Guangzhou, China

*Correspondence: Wenhai Zhao; *6177252@163.com;* Xiangxin Li; *13596109118@163.com*

Supporting Information

**S1 Table1. PRISMA 2020 checklist.**

| Section and Topic | Item # | Checklist item | Location where item  is reported |
| --- | --- | --- | --- |
| TITLE | | |  |
| Title | 1 | Identify the report as a systematic review. | P1 |
| ABSTRACT | | |  |
| Abstract | 2 | See the PRISMA 2020 for Abstracts checklist. | P1-2 |
| INTRODUCTION | | |  |
| Rationale | 3 | Describe the rationale for the review in the context of existing knowledge. | P2-3 |
| Objectives | 4 | Provide an explicit statement of the objective(s) or question(s) the review addresses. | P3 |
| METHODS | | |  |
| Eligibility criteria | 5 | Specify the inclusion and exclusion criteria for the review and how studies were grouped for the syntheses. | P4 |
| Information sources | 6 | Specify all databases, registers, websites, organisations, reference lists and other sources searched or consulted to identify studies. Specify the date when each source was last searched or consulted. | P4 |
| Search strategy | 7 | Present the full search strategies for all databases, registers and websites, including any filters and limits used. | P4 |
| Selection process | 8 | Specify the methods used to decide whether a study met the inclusion criteria of the review, including how many reviewers screened each record and each report retrieved, whether they worked independently, and if applicable, details of automation tools used in the process. | P4-5 |
| Data collection process | 9 | Specify the methods used to collect data from reports, including how many reviewers collected data from each report, whether they worked independently, any processes for obtaining or confirming data from study investigators, and if applicable, details of automation tools used in the process. | P4-5 |
| Data items | 10a | List and define all outcomes for which data were sought. Specify whether all results that were compatible with each outcome domain in each study were sought (e.g. for all measures, time points, analyses), and if not, the methods used to decide which results to collect. | P4-5 |
|  | 10b | List and define all other variables for which data were sought (e.g. participant and intervention characteristics, funding sources). Describe any assumptions made about any missing or unclear information. | P4-5 |
| Study risk of bias assessment | 11 | Specify the methods used to assess risk of bias in the included studies, including details of the tool(s) used, how many reviewers assessed each study and whether they worked independently, and if applicable, details of automation tools used in the process. | P5 |
| Effect measures | 12 | Specify for each outcome the effect measure(s) (e.g. risk ratio, mean difference) used in the synthesis or presentation of results. | P5 |
| Synthesis methods | 13a | Describe the processes used to decide which studies were eligible for each synthesis (e.g. tabulating the study intervention characteristics and comparing against the planned groups for each synthesis (item #5)). | P4-5 |
|  | 13b | Describe any methods required to prepare the data for presentation or synthesis, such as handling of missing summary statistics, or data conversions. | P5 |
|  | 13c | Describe any methods used to tabulate or visually display results of individual studies and syntheses. | P5 |
|  | 13d | Describe any methods used to synthesize results and provide a rationale for the choice(s). If meta-analysis was performed, describe the model(s), method(s) to identify the presence and extent of statistical heterogeneity, and software package(s) used. | P5 |
|  | 13e | Describe any methods used to explore possible causes of heterogeneity among study results (e.g. subgroup analysis, meta-regression). | P5 |
|  | 13f | Describe any sensitivity analyses conducted to assess robustness of the synthesized results. | P5 |
| Reporting bias assessment | 14 | Describe any methods used to assess risk of bias due to missing results in a synthesis (arising from reporting biases). | P5 |
| Section and Topic | Item # | Checklist item | Location where item  is reported |
| Certainty assessment | 15 | Describe any methods used to assess certainty (or confidence) in the body of evidence for an outcome. | P5 |
| RESULTS | | |  |
| Study selection | 16a | Describe the results of the search and selection process, from the number of records identified in the search to the number of studies included in the review, ideally using a flow diagram. | P6,figure 1 |
|  | 16b | Cite studies that might appear to meet the inclusion criteria, but which were excluded, and explain why they were excluded. | P6,figure 1 |
| Study characteristics | 17 | Cite each included study and present its characteristics. | P6 |
| Risk of bias in studies | 18 | Present assessments of risk of bias for each included study. | P6 |
| Results of individual studies | 19 | For all outcomes, present, for each study: (a) summary statistics for each group (where appropriate) and (b) an effect estimate and its precision (e.g. confidence/credible interval), ideally using structured tables or plots. | P7-9 |
| Results of syntheses | 20a | For each synthesis, briefly summarise the characteristics and risk of bias among contributing studies. | P6 |
|  | 20b | Present results of all statistical syntheses conducted. If meta-analysis was done, present for each the summary estimate and its precision (e.g. confidence/credible interval) and measures of statistical heterogeneity. If comparing groups, describe the direction of the effect. | P8-13 |
|  | 20c | Present results of all investigations of possible causes of heterogeneity among study results. | P8-13 |
|  | 20d | Present results of all sensitivity analyses conducted to assess the robustness of the synthesized results. | / |
| Reporting biases | 21 | Present assessments of risk of bias due to missing results (arising from reporting biases) for each synthesis assessed. | P8-13 |
| Certainty of evidence | 22 | Present assessments of certainty (or confidence) in the body of evidence for each outcome assessed. | P8-13 |
| DISCUSSION | | |  |
| Discussion | 23a | Provide a general interpretation of the results in the context of other evidence. | P13-17 |
|  | 23b | Discuss any limitations of the evidence included in the review. | P13-17 |
|  | 23c | Discuss any limitations of the review processes used. | P13-17 |
|  | 23d | Discuss implications of the results for practice, policy, and future research. | P13-17 |
| OTHER INFORMATION | | |  |
| Registration and protocol | 24a | Provide registration information for the review, including register name and registration number, or state that the review was not registered. | P3,CRD42023451297 |
|  | 24b | Indicate where the review protocol can be accessed, or state that a protocol was not prepared. | P3,CRD42023451297 |
|  | 24c | Describe and explain any amendments to information provided at registration or in the protocol. | / |
| Support | 25 | Describe sources of financial or non-financial support for the review, and the role of the funders or sponsors in the review. | P18 |
| Competing interests | 26 | Declare any competing interests of review authors. | P18 |
| Availability of data, code and other materials | 27 | Report which of the following are publicly available and where they can be found: template data collection forms; data extracted from included studies; data used for all analyses; analytic code; any other materials used in the review. | P18 |

S1 Table2. The search strategy of the PubMed database

| Number | Keywords of the Research |
| --- | --- |
| #1 | Arthritis [mh] |
| #2 | Arthritis [tiab] OR Arthritides [tiab] |
| #3 | #1 OR #2 |
| #4 | Colchicine [mh] |
| #5 | Colchicine [tiab] OR Colchicine, (R)-isomer [tiab] OR Colchicine, (+-)-isomer [tiab] |
| #6 | #4 OR #5 |
| #7 | Randomized controlled trial[pt] OR Controlled clinical trial[pt] |
| #8 | Randomized controlled trial[tiab] OR Controlled clinical trial[tiab] OR Randomized*[tiab] OR Randomly*[tiab] Random allocation[tiab] OR Trial[tiab] OR CCT[tiab] OR RCT[tiab] |
| #9 | #7 OR #8 |
| #10 | #3 AND #6 AND #9 |

Note: mh: MeSH; tiab: tittle/abstract; pt: publication type

S1 Table 3：Quality assessment of randomized controlled trials

| ID | Bias arising from the randomization process | Bias due to deviations from intended intervention | Bias due to missing outcome data | Bias in measurement of the outcome | Bias in selection of the reported result | Overall |
| --- | --- | --- | --- | --- | --- | --- |
| CR Davis 2021 | Low | Low | Low | Low | Low | Low |
| SK DAS 2002a | Low | Low | Low | Low | Low | Low |
| SK DAS 2002b | Low | Low | Low | Low | Low | Low |
| P Liu 2019 | Low | Low | Low | Low | Low | Low |
| YY Leung 2018 | Low | Low | Low | Low | Low | Low |
| S. Aran 2011 | Low | Low | Low | Low | Low | Low |
| A. Amirpour 2016 | some concern | Low | Low | Low | Low | some concern |
| Erden M 2012 | some concern | Low | Low | Low | Low | some concern |
| Borstad G 2004 | some concern | Low | some concerns | Low | Low | some concerns |
| McKendry R 1993 | Low | Low | Low | Low | Low | Low |
| Pascart T 2023 | Low | Low | Low | Low | Low | Low |

S1 Table 4：A table of all data extracted from the primary research sources

|  | colchicine | | | non-colchicine | | |
| --- | --- | --- | --- | --- | --- | --- |
| Study_ID | Mean | SD | Number | Mean | SD | Number |
| VAS | | | | | | |
| CR Davis 2021 | -8 | 20 | 32 | -18 | 21.4 | 32 |
| SK DAS 2002a | -2.7 | 2.7 | 19 | -2.2 | 3.3 | 17 |
| SK DAS 2002b | -0.6 | 0.1 | 19 | -0.1 | 0.1 | 20 |
| A. Amirpour 2016 | -2.2 | 2.6 | 32 | -1.2 | 2.4 | 30 |
| Erden M 2012 | -4 | 8.5 | 30 | -4 | 8 | 30 |
| WOMAC pain score | | | | | | |
| YY Leung 2018 | -13.7 | 20 | 54 | -16.8 | 18.9 | 55 |
| A. Amirpour 2016 | -4 | 4.6 | 32 | -3 | 4 | 30 |
| Erden M 2012 | -1 | 3 | 30 | -1 | 2.25 | 30 |
| WOMAC function score | | | | | | |
| YY Leung 2018 | -14.6 | 19 | 54 | -13.5 | 17.9 | 55 |
| A. Amirpour 2016 | -11 | 13 | 32 | -7 | 10 | 30 |
| Erden M 2012 | -2 | 8.3 | 30 | -2.5 | 6.5 | 30 |
| total WOMAC scale score | | | | | | |
| SK DAS 2002a | -8.6 | 13 | 19 | -1.5 | 18 | 17 |
| YY Leung 2018 | -13.1 | 18 | 54 | -13.9 | 17 | 55 |
| Erden M 2012 | -1 | 2.8 | 30 | -1 | 1.9 | 30 |
| Physician's global assessment score | | | | | | |
| SK DAS 2002a | -1.7 | 2.2 | 19 | 0.6 | 2.5 | 17 |
| S. Aran 2011 | 9.83 | 3.8 | 31 | 3.72 | 3.4 | 30 |
| Patient's global assessment score | | | | | | |
| 97SK DAS 2002a | 2.6 | 2.5 | 19 | -0.3 | 3.2 | 17 |
| S. Aran 2011 | 11.14 | 4.1 | 31 | 3.14 | 2.2 | 30 |
| YY Leung 2018 | -13.4 | 23 | 54 | -20.9 | 23 | 55 |
| ModHAQ score | | | | | | |
| SK DAS 2002a | -3 | 2.8 | 19 | -1.1 | 2.5 | 17 |
| SK DAS 2002b | -3.7 | 0.4 | 19 | -1 | 0.4 | 20 |
| YY Leung 2018 | -0.1 | 0.4 | 54 | -0.2 | 0.3 | 55 |
| A. Amirpour 2016 | -2 | 2 | 32 | -2 | 2.6 | 30 |
| Stiffness | | | | | | |
| A. Amirpour 2016 | -2 | 2 | 32 | -1 | 2 | 30 |
| Erden M 2012 | -1 | 1.3 | 30 | 0 | 0 | 30 |
| Adverse events | | | | | | |
|  | Yes | NO | Total Number | Yes | NO | Total Number |
| P Liu 2019 | 7 | 45 | 52 | 5 | 48 | 53 |
| Borstad, G2004 | 9 | 12 | 21 | 8 | 14 | 22 |
| CR Davis 2021 | 22 | 10 | 32 | 12 | 20 | 32 |
| SK DAS 2002a | 11 | 8 | 19 | 4 | 13 | 17 |
| SK DAS 2002b | 19 | 0 | 19 | 20 | 0 | 20 |
| YY Leung 2018 | 42 | 12 | 54 | 35 | 20 | 55 |
| S. Aran 2011 | 1 | 30 | 31 | 0 | 30 | 30 |
| A. Amirpour 2016 | 0 | 32 | 32 | 0 | 30 | 30 |

S1 Table 5：All studies identified in the literature search

| No. | Tittle |
| --- | --- |
| 1 | Janssens HJ, Lucassen P, Van de Laar FA, et al. Systemic corticosteroids for acute gout. Cochrane Database of Systematic Reviews 2008. DOI: 10.1002/14651858.CD005521.pub2. |
| 2 | Jones G, Crotty M and Brooks P. Interventions for treating psoriatic arthritis. Cochrane Database of Systematic Reviews 2000. DOI: 10.1002/14651858.CD000212. |
| 3 | McKenzie BJ, Wechalekar MD, Johnston RV, et al. Colchicine for acute gout. Cochrane Database of Systematic Reviews 2021. DOI: 10.1002/14651858.CD006190.pub3. |
| 4 | Moi JHY, Sriranganathan MK, Edwards CJ, et al. Lifestyle interventions for acute gout. Cochrane Database of Systematic Reviews 2013. DOI: 10.1002/14651858.CD010519.pub2. |
| 5 | Saenz A, Ausejo M, Shea B, et al. Pharmacotherapy for Behcet's syndrome. Cochrane Database of Systematic Reviews 1998. DOI: 10.1002/14651858.CD001084. |
| 6 | Seth R, Kydd ASR, Buchbinder R, et al. Allopurinol for chronic gout. Cochrane Database of Systematic Reviews 2014. DOI: 10.1002/14651858.CD006077.pub3. |
| 7 | Sivera F, Wechalekar MD, Andrés M, et al. Interleukin‐1 inhibitors for acute gout. Cochrane Database of Systematic Reviews 2014. DOI: 10.1002/14651858.CD009993.pub2. |
| 8 | van Durme C, Wechalekar MD, Landewé RBM, et al. Non‐steroidal anti‐inflammatory drugs for acute gout. Cochrane Database of Systematic Reviews 2021. DOI: 10.1002/14651858.CD010120.pub3. |
| 9 | Alten R, Bardin T, Bloch M, et al. Safety and efficacy of canakinumab in frequently flaring gouty arthritis patients who are contraindicated, intolerant or unresponsive to non-steroidal anti-inflammatory drugs and/or colchicine: results from 3 years follow-up. Annals of the rheumatic diseases 2015; 74: 544. Journal article; Conference proceeding. DOI: 10.1136/annrheumdis-2015-eular.2199. |
| 10 | Alten R, Bloch M, Bardin T, et al. Efficacy and safety of canakinumab vs triamcinolone acetonide in persistent or elderly gouty arthritis patients. Annals of the rheumatic disease 2013; 71. Journal article; Conference proceeding. DOI: 10.1136/annrheumdis-2012-eular.1080. |
| 11 | Alvarado JA, Lopez JM, Bardan AC, et al. Efficacy of atorvastatin versus colchicine in decreasing biomarkers of myocardial damage in patients with severe rheumatoid arthritis. Annals of the rheumatic diseases 2020; 79: 600‐601. Journal article; Conference proceeding. DOI: 10.1136/annrheumdis-2020-eular.2513. |
| 12 | Amarilyo G, Rothman D, Manthiram K, et al. Consensus treatment plans for periodic fever, aphthous stomatitis, pharyngitis and adenitis syndrome (PFAPA): a framework to evaluate treatment responses from the childhood arthritis and rheumatology research alliance (CARRA) PFAPA work group. Pediatric rheumatology online journal 2020; 18: 31. Journal article; Conference proceeding. DOI: 10.1186/s12969-020-00424-x. |
| 13 | Bardin T, So A, Alten R, et al. Efficacy and safety of canakinumab vs triamcinolone acetonide in patients with gouty arthritis unable to use nonsteroidal anti-inflammatory drugs and colchicine, and on stable urate lowering therapy (ULT) or unable to use ULT. Arthritis and rheumatism 2012; 64: S811‐S812. Journal article; Conference proceeding. DOI: 10.1002/art.37735. |
| 14 | Barry A, Helget LN, Androsenko M, et al. A Comparison of Gout Flares with the Initiation of Treat-to-Target Allopurinol and Febuxostat: a Post-hoc Analysis of a Randomized Multicenter Trial. Arthritis & rheumatology 2024. Journal article. DOI: 10.1002/art.42927. |
| 15 | Barry A, Helget LN, Androsenko M, et al. Comparison of Gout Flares With the Initiation of Treat-to-Target Allopurinol and Febuxostat: a Post-Hoc Analysis of a Randomized Multicenter Trial. Arthritis & rheumatology 2024. Journal article. DOI: 10.1002/art.42927. |
| 16 | BenEzra D, Cohen E, Chajek T, et al. Evaluation of conventional therapy versus cyclosporine A in Behçet's syndrome. Transplantation proceedings 1988; 20: 136‐143. Journal article. |
| 17 | Bi XJ, Chen RF, Ren B, et al. Clinical research of Bixie Decoction in treating acute gouty arthritis. Acta chinese medicine and pharmacology [zhong yi yao xue bao] 2007; 35: 49‐50. Journal article. |
| 18 | Black S, McKee N, McKnight J, et al. The TICOG Study: tight Control of Gout-A Randomized, Controlled Trial of Targeted versus Conventional Treatment for Gout Including Ultrasonography. Arthritis & rheumatology 2022; 74: 3122‐3123. Journal article; Conference proceeding. DOI: 10.1002/art.42355. |
| 19 | Borstad GC, Bryant LR, Abel MP, et al. Colchicine for prophylaxis of acute flares when initiating allopurinol for chronic gouty arthritis. Journal of rheumatology 2004; 31: 2429‐2432. Journal article. |
| 20 | Botson J, Peloso PM, Obermeyer K, et al. Pegloticase response improvement by co-treatment with methotrexate: results from the mirror open-label clinical trial in patients with uncontrolled gout. Annals of the rheumatic diseases 2020; 79: 446. Journal article; Conference proceeding. DOI: 10.1136/annrheumdis-2020-eular.3932. |
| 21 | Brown JP, So A, Dikranian A, et al. Long-term efficacy and safety of canakinumab versus triamcinolone acetonide in acute gouty arthritis patients. Arthritis and rheumatism 2011; 63. Journal article; Conference proceeding. |
| 22 | Calgüneri M, Kiraz S, Ertenli I, et al. The effect of prophylactic penicillin treatment on the course of arthritis episodes in patients with Behçet's disease. A randomized clinical trial. Arthritis and rheumatism 1996; 39: 2062‐2065. Journal article. DOI: 10.1002/art.1780391216. |
| 23 | Cao JJ, Yang WJ and Cao Y. Electroacupuncture and moxibustion combined with cupping therapy in the treatment of acute gouty arthritis for 60 cases. Chinese medicine modern distance education of china [zhong guo zhong yi yao xian dai yuan chen jiao yu] 2016; 14: 110‐112. Journal article. |
| 24 | Chhibar PS and Ehresmann G. Anakinra as a successful treatment of idiopathic recurrent pericarditis: taper or not to taper? case series at the university of Southern California. Annals of the rheumatic diseases 2017; 76: 421‐422. Journal article; Conference proceeding. DOI: 10.1136/annrheumdis-2017-eular.1141. |
| 25 | ChiCtr. A multicenter, randomized, double-blind, parallel controlled clinical trial on the efficacy and safety of Chuanhu Qutong Granules in the treatment of acute gouty arthritis. https://trialsearchwhoint/Trial2aspx?TrialID=ChiCTR2100042564 2021. Trial registry record. |
| 26 | ChiCtr. Effect of Wuweixiaodu Decoction on Clinical Efficacy and Inflammatory Factors of Acute Gouty Arthritis. https://trialsearchwhoint/Trial2aspx?TrialID=ChiCTR2100047807 2021. Trial registry record. |
| 27 | ChiCtr. Immediate analgesic effects of cheek acupuncture for acute gouty arthritis: protocol for a randomised controlled trial. https://trialsearchwhoint/Trial2aspx?TrialID=ChiCTR2100054396 2021. Trial registry record. |
| 28 | ChiCtr. Randomized parallel controlled clinical trial of Qinpi Tongfeng decoction combined with bloodletting in the treatment of acute gouty arthritis (damp heat accumulation syndrome). https://trialsearchwhoint/Trial2aspx?TrialID=ChiCTR2100048836 2021. Trial registry record. |
| 29 | ChiCtr. Clinical trial of Aixite medicinal charcoal tablets in preventing acute attack during the treatment of primary gout and reducing uric acid. https://trialsearchwhoint/Trial2aspx?TrialID=ChiCTR2200066023 2022. Trial registry record. |
| 30 | ChiCtr. Randomized, controlled, multicenter clinical study of rebamipide in the prevention of gout attack. https://trialsearchwhoint/Trial2aspx?TrialID=ChiCTR2300078984 2023. Trial registry record. |
| 31 | Couderc M, Mathieu S, Glace B, et al. Efficacy of anakinra in pseudogout: report of 3 cases. Annals of the rheumatic disease 2013; 71. Journal article; Conference proceeding. DOI: 10.1136/annrheumdis-2012-eular.1067. |
| 32 | Ctis. Randomized controlled trial in patients on long-term colchicine with colchicine-resistant familial Mediterranean fever (FMF) to evaluate the efficacy of on-demand Anakinra treatment for painful attacks in patients who refuse continuous daily therapy (KIN-ATTACK-FMF). https://trialsearchwhoint/Trial2aspx?TrialID=CTIS2023-506721-11-00 2023. Trial registry record. |
| 33 | Dalbeth N, Saag KG, Palmer WE, et al. Effects of Febuxostat in Early Gout: a Randomized, Double-Blind, Placebo-Controlled Study. Arthritis & rheumatology 2017; 69: 2386‐2395. Journal article. DOI: 10.1002/art.40233. |
| 34 | Davis C, Ruediger C, Dyer K, et al. Colchicine is not effective for reducing osteoarthritic hand pain compared to placebo: a randomised, placebo-controlled trial (COLAH). Annals of the rheumatic diseases 2020; 79: 797‐798. Journal article; Conference proceeding. DOI: 10.1136/annrheumdis-2020-eular.4040. |
| 35 | Demiroglu H, Ozcebe OI, Barista I, et al. Interferon alfa-2b, colchicine, and benzathine penicillin versus colchicine and benzathine penicillin in Behçet's disease: a randomised trial. Lancet (london, england) 2000; 355: 605‐609. Journal article; Retracted Publication. DOI: 10.1016/S0140-6736(99)05131-4. |
| 36 | Deng W. Fortifying the spleen and detoxification drain turbidity therapy in treating gouty arthritis. Journal of chinese medicinal materials [zhong yao cai] 2003; 26: 466‐467. Journal article. |
| 37 | Dominguez-Casas LC, Calvo-Rio V, Blanco R, et al. Anti-IL6-R tocilizumab in refractory uveitis associated with Behcet's disease. Multicenter study of 11 patients. Arthritis & rheumatology 2016; 68: 3939‐3941. Journal article; Conference proceeding. DOI: 10.1002/art.39977. |
| 38 | Dorfler H. Treatment of acute attacks of gout with acemetacin. Die Therapiewoche 1985; 35: 1161‐1166. Journal article. |
| 39 | Duzova A, Bakkaloglu A, Besbas N, et al. Role of A-SAA in monitoring subclinical inflammation and in colchicine dosage in familial Mediterranean fever. Clinical and experimental rheumatology 2003; 21: 509‐514. Journal article. |
| 40 | Euctr DE. Study to assess serum uric acid lowering effects and safety of lesinurad compared to placebo in patients who are intolerant or have a contraindication to allopurinol or febuxostat. https://trialsearchwhoint/Trial2aspx?TrialID=EUCTR2011-003756-39-DE 2012. Trial registry record. |
| 41 | Euctr DE. Tocilizumab for the Treatment of Familial Mediterranean Fever. https://trialsearchwhoint/Trial2aspx?TrialID=EUCTR2016-004505-13-DE 2017. Trial registry record. |
| 42 | Euctr EE. A randomized, controlled study of ACZ885 (canakinumab) on the treatment and prevention of gout flares in patients with frequent flares for whom NSAIDs and/ or colchicine are contraindicated, not tolerated or ineffective - H2357. https://trialsearchwhoint/Trial2aspx?TrialID=EUCTR2009-017802-35-EE 2010. Trial registry record. |
| 43 | Euctr FR. An adaptive dose-ranging, multi-center, single-blind, double-dummy, active-controlled trial to determine the target dose of canakinumab (ACZ885) in the treatment of acute flares in gout patients who are refractory or contraindicated to NSAIDs and/or colchicine. https://trialsearchwhoint/Trial2aspx?TrialID=EUCTR2008-004666-61-FR 2008. Trial registry record. |
| 44 | Euctr GR. A clinical study on the therapeutic efficacy and safety of Febuxostat and Allopurinol on serum urate concentration in subjects suffering from hyperuricemia and gout. https://trialsearchwhoint/Trial2aspx?TrialID=EUCTR2012-001858-25-GR 2012. Trial registry record. |
| 45 | Euctr NL. Research into continuation or cessation of urate lowering therapies in gout patients in remission. https://trialsearchwhoint/Trial2aspx?TrialID=EUCTR2020-005730-15-NL 2021. Trial registry record. |
| 46 | Euctr PL. A randomized, double-blind, double-dummy, active controlled study of ACZ885 (canakinumab) on the treatment and prevention of gout flares in patients with frequent flares, for whom NSAIDs and/or colchicine are contraindicated, not tolerated or ineffective. https://trialsearchwhoint/Trial2aspx?TrialID=EUCTR2010-024172-26-PL 2011. Trial registry record. |
| 47 | Euctr SE. A randomized, controlled study of ACZ885 (canakinumab) on the treatment and prevention of gout flares in patients with frequent flares, who have pre-defined co-morbidities and for whom NSAIDs and colchicine are contraindicated, not tolerated or ineffective - H2356. https://trialsearchwhoint/Trial2aspx?TrialID=EUCTR2009-015018-23-SE 2009. Trial registry record. |
| 48 | Finckh A, Mc Carthy GM, Madigan A, et al. Methotrexate in chronic-recurrent calcium pyrophosphate deposition disease: no significant effect in a randomized crossover trial. Arthritis research & therapy 2014; 16: 458. Journal article. DOI: 10.1186/s13075-014-0458-4. |
| 49 | Guillot X, Tordi N, Laheurte C, et al. Local cryotherapy (pulsed CO2 or ice) decreases IL-6, IL-1β and VEGF synovial levels in knee arthritis. Annals of the rheumatic diseases 2016; 75: 896. Journal article; Conference proceeding. DOI: 10.1136/annrheumdis-2016-eular.1699. |
| 50 | Hannequin JR. Efficacy of Arthrotec in the treatment of rheumatoid arthritis. Scandinavian journal of rheumatology Supplement 1992; 96: 7‐14. Journal article. |
| 51 | Hashkes P and Huang B. The familial mediterranean fever (FMF) 50 score: does it work in a controlled clinical trial? Re-analysis of the trial of rilonacept for patients with colchicine resistant or intolerant FMF. Pediatric rheumatology 2015; 13: 234DUMMY. Journal article; Conference proceeding. |
| 52 | Hashkes PJ and Huang B. The familial Mediterranean fever (FMF) 50 score: does it work in a controlled clinical trial? Re-analysis of the trial of rilonacept for patients with colchicine-resistant or intolerant FMF. Israel Medical Association journal 2015; 17: 137‐140. Journal article. |
| 53 | He Y, Dai C, Shen J, et al. Effect of Baihu and Guizhi decoction in acute gouty arthritis: study protocol for a randomized controlled trial. Trials 2022; 23: 317. Journal article. DOI: 10.1186/s13063-022-06194-z. |
| 54 | Higginbottom A, Blackburn S, Campbell L, et al. Celebrating ten years of successful patient involvement in research of inflammatory conditions. Annals of the rheumatic diseases 2017; 76: 1552. Journal article; Conference proceeding. DOI: 10.1136/annrheumdis-2017-eular.3144. |
| 55 | Hill EM, Sky K, Sit M, et al. Does starting allopurinol prolong acute treated gout? A randomized clinical trial. Journal of clinical rheumatology 2015; 21: 120‐125. Journal article. DOI: 10.1097/RHU.0000000000000235. |
| 56 | Hosoya T, Sasaki T, Hashimoto H, et al. Clinical efficacy and safety of topiroxostat in Japanese male hyperuricemic patients with or without gout: an exploratory, phase 2a, multicentre, randomized, double-blind, placebo-controlled study. Journal of clinical pharmacy and therapeutics 2016; 41: 298‐305. Journal article. DOI: 10.1111/jcpt.12392. |
| 57 | Isrctn. Chuanhu anti-gout mixture in therapy of acute gouty arthritis. https://trialsearchwhoint/Trial2aspx?TrialID=ISRCTN65219941 2013. Trial registry record. |
| 58 | Itmctr. The anti-inflammatory effects of Mahajak oil in patients with acute gout attack; Pilot study. https://trialsearchwhoint/Trial2aspx?TrialID=ITMCTR2024000032 2024. Trial registry record. |
| 59 | Janssens HJ, Janssen M, van de Lisdonk EH, et al. Use of oral prednisolone or naproxen for the treatment of gout arthritis: a double-blind, randomised equivalence trial. Lancet (london, england) 2008; 371: 1854‐1860. Journal article. DOI: 10.1016/S0140-6736(08)60799-0. |
| 60 | Jia E, Hu S, Geng H, et al. Zhengqing fengtongning sustained-release tablets prevents gout flares in the process of ULT: a randomized, positive control, double-blind, double-simulation, multicenter trial. Medicine 2022; 101: e29199. Journal article. DOI: 10.1097/MD.0000000000029199. |
| 61 | Karimzadeh H, Nazari J, Mottaghi P, et al. Different duration of colchicine for preventing recurrence of gouty arthritis. Journal of research in medical sciences 2006; 11: 104‐107. Journal article. |
| 62 | Kct. A Multi-center, Randomized, Double-blind, Placebo-controlled Pilot Study to Evaluate the Efficacy and Safety of Diacerein for Prophylaxis in Patients with Gout who Have Difficulties to Use Colchicine for Prophylaxis. https://trialsearchwhoint/Trial2aspx?TrialID=KCT0007719 2022. Trial registry record. |
| 63 | Khanna P and Beal R. PHASE 2A, RANDOMIZED, DOUBLE-BLIND, PLACEBO-CONTROLLED STUDY of the EFFICACY and SAFETY of A TRANSDERMAL ALKALINIZING and PAIN-RELIEVING TREATMENT for REDUCING PAIN ASSOCIATED with AN ACUTE GOUT FLARE. Annals of the rheumatic diseases 2022; 81: 112. Journal article; Conference proceeding. DOI: 10.1136/annrheumdis-2022-eular.4909. |
| 64 | Khanna P and Beal R. The Impact of Topically Applied pH Modulator on Acute Inflammatory Pain, Serum Calcium, and C-Reactive Protein (CRP) during an Acute Gout Flare-a Phase 2a Randomized, Double-Blind, Placebo-Controlled Study. Arthritis & rheumatology 2022; 74: 3529‐3531. Journal article; Conference proceeding. DOI: 10.1002/art.42355. |
| 65 | Krishnan E, Pandya BJ, Lingala B, et al. Hyperuricemia and untreated gout are poor prognostic markers among those with a recent acute myocardial infarction. Arthritis research & therapy 2012; 14: R10. Journal article. DOI: 10.1186/ar3684. |
| 66 | Kulaksiz B, Egeli BH, Aydin O, et al. Is exon 2 associated with FMF or a new disease. Arthritis & rheumatology 2019; 71: 2162‐2164. Journal article; Conference proceeding. DOI: 10.1002/art.41108. |
| 67 | Kuppuraju R, May P, Millliken T, et al. TREATMENT OF GOUT AND ITS EFFECTS ON METABOLIC SYNDROME. Annals of the rheumatic diseases 2023; 82: 521‐522. Journal article; Conference proceeding. DOI: 10.1136/annrheumdis-2023-eular.3206. |
| 68 | Laosuksri P, Phrintrakul N, Gumtorntip W, et al. Non-loading versus loading low-dose colchicine in acute crystal-associated arthritis: a double-blinded randomized controlled study. International journal of rheumatic diseases 2023; 26: 2478‐2488. Journal article. DOI: 10.1111/1756-185X.14943. |
| 69 | Leccese P, Ozguler Y, Christensen R, et al. A systematic literature review on the treatment of skin, mucosa and joint involvement of beh��et's syndrome informing the eular recommendations for the management of behçet's syndrome. Annals of the rheumatic diseases 2016; 75: 798. Journal article; Conference proceeding. DOI: 10.1136/annrheumdis-2016-eular.5609. |
| 70 | Leung YY, Huebner JL, Wong B, et al. Association of synovial fluid inflammatory biomarkers and knee pain. Osteoarthritis and cartilage 2016; 24: S73. Journal article; Conference proceeding. |
| 71 | Li CD, Gao L, You GS, et al. Infrared warmed acupuncture in treating acute gouty arthritis. Nei mongol journal of traditional chinese medicine [nei meng GU zhong yi yao] 2008; 22: 38‐39. Journal article. |
| 72 | Li FQ. Research of treatment control for elderly primary chronic gouty arthritis. China jounal of clinical medicine [zhong guo lin chuang yi yao yan jiu za zhi] 2007; 8: 6‐8. Journal article. |
| 73 | Li JS, Guo FL, Yu JH, et al. Observation on therapeutic efects of moxibustion in patients with acute gouty arthritis. Journal of chinese modem traditional chinese medicine [zhong guo xiang dai zhong yi xue za zhi] 2006; 2: 97‐99. Journal article. |
| 74 | Liang H, Wu Y, Zhang W, et al. Efficacy and Safety of Acupuncture Combined with Herbal Medicine in Treating Gouty Arthritis: meta-Analysis of Randomized Controlled Trials. Evidence-based complementary and alternative medicine : eCAM 2021; 2021: 8161731. Journal article. DOI: 10.1155/2021/8161731. |
| 75 | Lin N, Dai Q, Zhang Y, et al. Chinese classical decoction Wuwei Xiaodu Drink alleviates gout arthritis by suppressing NLRP3-Mediated inflammation. Frontiers in pharmacology 2024; 15. Journal article. DOI: 10.3389/fphar.2024.1388753. |
| 76 | Liu P, Chen Y, Wang B, et al. Expression of microRNAs in the plasma of patients with acute gouty arthritis and the effects of colchicine and etoricoxib on the differential expression of microRNAs. Archives of medical science 2019; 15: 1047‐1055. Journal article. DOI: 10.5114/aoms.2018.75502. |
| 77 | Liu Y, Li ZC, Chen JB, et al. Therapeutic efficacy of small doses of colchicine combined with glucocorticoid for acute gouty arthritis. Medical journal of chinese people's liberation army 2015; 40: 652‐655. Journal article. DOI: 10.11855/j.issn.0577-7402.2015.08.10. |
| 78 | Lu H, Liu W, Fan Y, et al. Efficacy and Safety of Qinpi Tongfeng Formula Combined with Bloodletting Therapy in the Treatment of Acute Gouty Arthritis: a Study Protocol for a Randomized Controlled Trial. Evidence-based complementary and alternative medicine : eCAM 2022; 2022: 3147319. Journal article. DOI: 10.1155/2022/3147319. |
| 79 | McKendry RJ, Kraag G, Seigel S, et al. Therapeutic value of colchicine in the treatment of patients with psoriatic arthritis. Annals of the rheumatic diseases 1993; 52: 826‐828. Journal article. DOI: 10.1136/ard.52.11.826. |
| 80 | Navarra S, Rubin BR, Yu Q, et al. Association of baseline disease and patient characteristics with response to etoricoxib and indomethacin for acute gout. Current medical research and opinion 2007; 23: 1685‐1691. Journal article. DOI: 10.1185/030079907x210750. |
| 81 | Nct. Colchicine Or Naproxen Treatment for ACute gouT. https://clinicaltrialsgov/show/NCT01994226 2013. Trial registry record. |
| 82 | Nct. The Comparison of the Efficacy of Once and Twice Daily Colchicine Dosage in Pediatric Patients With FMF. https://clinicaltrialsgov/show/NCT02602028 2015. Trial registry record. |
| 83 | Nct. Trial of Colchicine Versus Prednisone for the Treatment of Acute CPPD Arthritis. https://clinicaltrialsgov/show/NCT03128905 2017. Trial registry record. |
| 84 | Nct. Clinical Trial to Evaluate the Efficacy of a Dyslipidemic Therapy in Mexican Population. https://clinicaltrialsgov/show/NCT03696940 2018. Trial registry record. |
| 85 | Nct. Atorvastatin vs Colchicine in Decrease of Troponin I of High Sensitivity in Patients With Rheumatoid Arthritis. https://clinicaltrialsgov/show/NCT04056039 2019. Trial registry record. |
| 86 | Nct. Anti Inflammatory Effects of Local Cryotherapy in Knee Arthritis. https://clinicaltrialsgov/show/NCT03850392 2019. Trial registry record. |
| 87 | Nct. Prednisolone Versus Colchicine for Acute Gout in Primary Care. https://clinicaltrialsgov/ct2/show/NCT05698680 2022. Trial registry record. |
| 88 | Nct. A Phase Ib/II Study of Recombinant Anti-IL-1β Humanized Monoclonal Antibody Injection in Chinese Participants With Acute Gout. https://clinicaltrialsgov/show/NCT05588908 2022. Trial registry record. |
| 89 | Nct. Tight Control of Gouty Arthritis Compared to Usual Care. https://clinicaltrialsgov/ct2/show/NCT05507723 2022. Trial registry record. |
| 90 | Nct. An Innovative Chinese Herbal Formula for the Treatment of Gout. https://clinicaltrialsgov/show/NCT05499312 2022. Trial registry record. |
| 91 | Nct. Safety and Efficacy of Genakumab for Injection in Patients With Gout Flare. https://clinicaltrialsgov/show/NCT05936268 2023. Trial registry record. |
| 92 | Nct. A Study of Recombinant Anti-IL-1β Humanized Monoclonal Antibody Injection in Chinese Participants With Acute Gout. https://clinicaltrialsgov/ct2/show/NCT06169891 2023. Trial registry record. |
| 93 | Nct. Colchicine in Acutely Decompensated HFREF. https://clinicaltrialsgov/ct2/show/NCT06286423 2024. Trial registry record. |
| 94 | Nilsson F, Norberg B, Frederiksen B, et al. The role of the MA-sensitive leukocyte chemotaxis in rheumatoid arthritis. A randomized double-blind clinical trial of griseofulvin treatment. Scandinavian journal of rheumatology 1983; 12: 113‐118. Journal article. DOI: 10.3109/03009748309102895. |
| 95 | Nl O. A randomized, controlled study of ACZ885 (canakinumab) on the treatment and prevention of gout flares in patients with frequent flares for whom NSAIDs and/ or colchicine are contraindicated, not tolerated or ineffective (CACZ885H2357). https://trialsearchwhoint/Trial2aspx?TrialID=NL-OMON35054 2010. Trial registry record. |
| 96 | Nl O. Anakinra versus treatment as usual in the treatment of acute gout. https://trialsearchwhoint/Trial2aspx?TrialID=NL-OMON47178 2015. Trial registry record. |
| 97 | Nl O. The Effect of Intensive Urate Lowering Therapy (ULT) with Febuxostat in Comparison with Allopurinol on Cardiovascular Risk in Patients with Gout Using Surrogate Markers: a Randomized, Controlled Trial. https://trialsearchwhoint/Trial2aspx?TrialID=NL-OMON43921 2016. Trial registry record. |
| 98 | Pascart T, Robinet P, Ottaviani S, et al. Evaluating the safety and short-term equivalence of colchicine versus prednisone in older patients with acute calcium pyrophosphate crystal arthritis (COLCHICORT): an open-label, multicentre, randomised trial. The lancet Rheumatology 2023; 5: e523‐e531. Journal article. DOI: 10.1016/S2665-9913(23)00165-0. |
| 99 | Pascart T, Robinet P, Ottaviani S, et al. Colchicine or Prednisone for the Treatment of Acute Calcium Pyrophosphate Deposition Arthritis: a Multicenter Randomized Controlled Trial. Arthritis & rheumatology 2022; 74: 3131‐3134. Journal article; Conference proceeding. DOI: 10.1002/art.42355. |
| 100 | Perez-Ruiz F, Ruibal Escribano A, Alonso Santamaria C, et al. Gout in the elderly. Seminarios de la fundacion espanola de reumatologia 2002; 3: 4‐12. Journal article. |
| 101 | Polat A, Acikel C, Sozeri B, et al. Comparison of the efficacy of once- and twice-daily colchicine dosage in pediatric patients with familial Mediterranean fever--a randomized controlled noninferiority trial. Arthritis research & therapy 2016; 18: 85. Journal article. DOI: 10.1186/s13075-016-0980-7. |
| 102 | Ramesh R, Sriram S, Kumudha M, et al. Interleukin 1B levels in acute gout-before and after colchicine-a randomised prospective interventional study. International journal of rheumatic diseases 2015; 18: 17‐18. Journal article; Conference proceeding. |
| 103 | Ramos-Bello D, Alvarez-Quiroga C, Barragan-Pickens GA, et al. Effect of methotrexate (MTX), and MTX plus colchicine (CCH) on the expression and activity of NLRP3 inflammasome in patients with early rheumatoid arthritis (RA). Arthritis & rheumatology 2017; 69. Journal article; Conference proceeding. |
| 104 | Ren XY. Curative effect of Baihu Ramuli Cinamomi Decoction on acute gouty arthritis in 20 cases. Modern hospital [xian dai yi yuan] 2005; 5: 65. Journal article. |
| 105 | Richter A, Truthmann J, Hummers E, et al. Prednisolone Versus Colchicine for Acute Gout in Primary Care: statistical analysis plan for the pragmatic, multicenter, randomized, and double-blinded COPAGO non-inferiority trial. Trials 2024; 25: 229. Journal article. DOI: 10.1186/s13063-024-08066-0. |
| 106 | Saag K, So A, Khanna P, et al. A randomized, phase 2 study evaluating the efficacy and safety of Anakinra in difficult-to-treat acute gouty arthritis: the Anago study. Arthritis & rheumatology 2019; 71: 2136‐2138. Journal article; Conference proceeding. DOI: 10.1002/art.41108. |
| 107 | Saag K, So A, Khanna P, et al. A randomized, phase 2 study evaluating the efficacy and safety of anakinra in difficultto-treat acute gouty arthritis: the Anago study. Annals of the rheumatic diseases 2020; 79: 442. Journal article; Conference proceeding. DOI: 10.1136/annrheumdis-2020-eular.3766. |
| 108 | Samuels J, Bomfim F, Toprover M, et al. Colchicine for treatment of Osteoarthritis of the knee (CLOAK)—A phase 2, double-blind, placebo-controlled trial. Osteoarthritis and cartilage 2020; 28: S498. Journal article; Conference proceeding. DOI: 10.1016/j.joca.2020.02.781. |
| 109 | Samuels J, Pillinger M, Toprover M, et al. CoLchicine for Treatment of OsteoArthritis of the Knee (CLOAK)-A Double-blind, Placebo-controlled Trial. Arthritis & rheumatology 2022; 74: 3754‐3756. Journal article; Conference proceeding. DOI: 10.1002/art.42355. |
| 110 | Sandhu T, Tieng A, Chilimuri S, et al. A case control study to evaluate the impact of colchicine on patients admitted to the hospital with moderate to severe covid-19 infection. The Canadian journal of infectious diseases & medical microbiology = Journal canadien des maladies infectieuses et de la microbiologie medicale 2020; 2020: 8865954. Journal article. DOI: 10.1155/2020/8865954. |
| 111 | Satpanich P and Manavathongchai S. Early versus late allopurinol initiation in an acute gout attack: a randomized controlled trial (ELAG). International journal of rheumatic diseases 2020; 23: 178‐179. Journal article; Conference proceeding. DOI: 10.1111/1756-185X.13985. |
| 112 | Schlesinger N, Alten R, Bardin T, et al. Effect of canakinumab vs triamcinolone acetonide for treatment of gouty arthritis in patients who are unable to use NSAIDs and colchicine or with severe gouty arthritis. Annals of the rheumatic disease 2013; 71. Journal article; Conference proceeding. DOI: 10.1136/annrheumdis-2012-eular.2826. |
| 113 | Schlesinger N, Bardin T, Bloch M, et al. A 3-year follow-up study of canakinumab in frequently flaring gouty arthritis patients, contraindicated, intolerant, or unresponsive to nonsteroidal anti-inflammatory drugs and/or colchicine. Arthritis & rheumatology 2015; 67. Journal article; Conference proceeding. DOI: 10.1002/art.39448. |
| 114 | Schlesinger N, Brown JP, Bardin T, et al. Comparison of pain intensity, incidence of new flares, safety and tolerability of canakinumab vs triamcinolone acetonide in gouty arthritis patients with cardiovascular diseases or with cardiovascular risk factors. Arthritis and rheumatism 2011; 63. Journal article; Conference proceeding. |
| 115 | Schlesinger N, De Meulemeester M, Pikhlak A, et al. Canakinumab relieves symptoms of acute flares and improves health-related quality of life in patients with difficult-to-treat Gouty Arthritis by suppressing inflammation: results of a randomized, dose-ranging study. Arthritis research & therapy 2011; 13: R53. Journal article. DOI: 10.1186/ar3297. |
| 116 | Schlesinger N, Detry MA, Holland BK, et al. Local ice therapy during bouts of acute gouty arthritis. Journal of rheumatology 2002; 29: 331‐334. Journal article. |
| 117 | Schlesinger N, Lin H, De Meulemeester M, et al. Efficacy of canakinumab (ACZ885), a fully human anti-interleukin-1beta monoclonal antibody, in the prevention of flares in gout patients initiating allopurinol therapy. Rheumatology 2011; 50: iii85‐iii86. Journal article; Conference proceeding. |
| 118 | Seideman P, Fjellner B and Johannesson A. Psoriatic arthritis treated with oral colchicine. Journal of rheumatology 1987; 14: 777‐779. Journal article. |
| 119 | Sharma A, Provenzale D, McKusick A, et al. Interstitial pneumonitis after low-dose methotrexate therapy in primary biliary cirrhosis. Gastroenterology 1994; 107: 266‐270. Journal article. DOI: 10.1016/0016-5085(94)90085-x. |
| 120 | So A, Alten R, Schumacher HR, et al. Inflammation suppression over 24 weeks in patients with gouty arthritis: results from two phase-III core and extension studies comparing canakinumab with triamcinolone acetonide. Arthritis and rheumatism 2011; 63. Journal article; Conference proceeding. |
| 121 | So A, De Meulemeester M, Pikhlak A, et al. Canakinumab (ACZ885) relieves pain and controls inflammation rapidly in patients with difficult-to-treat gouty arthritis: comparison with triamcinolone acetonide. Rheumatology 2011; 50: iii32. Journal article; Conference proceeding. |
| 122 | So A, De Meulemeester M, Pikhlak A, et al. Rapid improvement in health-related quality of life in gouty arthritis patients treated with canakinumab (ACZ885) compared to triamcinolone acetonide. Rheumatology 2011; 50: iii85. Journal article; Conference proceeding. |
| 123 | So A, De Meulemeester M, Pikhlak A, et al. Canakinumab for the treatment of acute flares in difficult-to-treat gouty arthritis: results of a multicenter, phase II, dose-ranging study. Arthritis and rheumatism 2010; 62: 3064‐3076. Journal article. DOI: 10.1002/art.27600. |
| 124 | Sunkureddi P, Schlesinger N, Kiechle T, et al. Effect of serum urate level on prevention of flare in acute gouty arthritis patients with canakinumab. Annals of the rheumatic disease 2013; 71. Journal article; Conference proceeding. DOI: 10.1136/annrheumdis-2012-eular.1079. |
| 125 | Sunkureddi P, Toth E, Brown J, et al. Canakinumab pre-filled syringe vs triamcinolone acetonide in the treatment of acute gouty arthritis attacks: results from a post-hoc analysis in difficult-to-treat patients. Annals of the rheumatic diseases 2014; 73. Journal article; Conference proceeding. DOI: 10.1136/annrheumdis-2014-eular.1846. |
| 126 | Sunkureddi P, Toth E, Brown JP, et al. Efficacy and safety of canakinumab pre-filled syringe versus triamcinolone acetonide in acute gouty arthritis patients. Arthritis and rheumatism 2013; 65: S498. Journal article; Conference proceeding. DOI: 10.1002/art.38216. |
| 127 | Tctr. Efficacy and safety of NSAIDs spray for pain relieving in patients with acute gouty arthritis : a randomized control trial. https://trialsearchwhoint/Trial2aspx?TrialID=TCTR20230208012 2023. Trial registry record. |
| 128 | Wang H, Chen ST, Ding XJ, et al. Efficacy and safety of Huzhang Granule, a compound Chinese herbal medicine, for acute gouty arthritis: a double-blind, randomized controlled trial. Journal of integrative medicine 2024. Journal article. DOI: 10.1016/j.joim.2024.03.008. |
| 129 | Wang H, Chen ST, Ding XJ, et al. Efficacy and safety of Huzhang Granule, a compound Chinese herbal medicine, for acute gouty arthritis: a double-blind, randomized controlled trial. Journal of integrative medicine 2024; 22: 270‐278. Journal article. DOI: 10.1016/j.joim.2024.03.008. |
| 130 | Wang Y, Wang L, Li E, et al. Chuanhu anti-gout mixture versus colchicine for acute gouty arthritis: a randomized, double-blind, double-dummy, non-inferiority trial. International journal of medical sciences 2014; 11: 880‐885. Journal article. DOI: 10.7150/ijms.9165. |
| 131 | Wu HX. Qingre Xiezhuo Quyu Decoction combined with acupuncture in the treatment of acute episode of gout for 35 cases. Chinese medicine modern distance education of china [zhong guo zhong yi yao xian dai yuan cheng jiao yu] 2015; 13: 75‐76. Journal article. |
| 132 | Wu YQ, Hu JH, Zhao ZY, et al. Clinical observation on senile patients with acute gouty arthritis treated by acupoint application. Journal of the American Geriatrics Society 2014; 62: S381. Journal article; Conference proceeding. DOI: 10.1111/jgs.13075. |
| 133 | Yang DH, Chen HC and Wei J-C. Early lowering of serum uric acid levels in the treatment of gouty arthritis with an acute attack. International journal of rheumatic diseases 2018; 21: 218. Journal article; Conference proceeding. DOI: 10.1111/1756-185X.13361. |
| 134 | Yang DH, Chen HC and Wei JC. Early urate-lowering therapy in gouty arthritis with acute flares: a double-blind placebo controlled clinical trial. European journal of medical research 2023; 28: 10. Journal article. DOI: 10.1186/s40001-022-00982-8. |
| 135 | Yang ZH, Wang HM, Cui Q, et al. Clinical analysis of Jianpi Qingre Lishi Tongluo Formula for treating acute gouty arthritis in 60 cases. Modern medicine journal of china [zhong guo xian dai yi yao za zhi] 2007; 9: 82‐83. Journal article. |
| 136 | Yu J, Li L, Liu J, et al. Influence of intervention treatment by "heat-clearing and diuresis-promoting" prescription on NALP3, an inflammatory factor in acute gouty arthritis. Journal of orthopaedic surgery and research 2022; 17: 162. Journal article. DOI: 10.1186/s13018-022-03046-z. |
| 137 | Yu Z and Li JG. Clinical observation of combination of traditional Chinese and western medicine in the treatment of acute gouty arthritis in 40 cases. Chinese community doctors[zhong guo she qu yi shi] 2016; 32: 93.95. Journal article. |
| 138 | Yurdakul S, Mat C, Tüzün Y, et al. A double-blind trial of colchicine in Behçet's syndrome. Arthritis and rheumatism 2001; 44: 2686‐2692. Journal article. DOI: 10.1002/1529-0131(200111)44:11<2686::aid-art448>3.0.co;2-h. |
| 139 | Zhang Y, Pan R, Xu Y, et al. Treatment of refractory gout with TNF-α antagonist etanercept combined with febuxostat. Annals of palliative medicine 2020; 9: 4332‐4338. Journal article. DOI: 10.21037/apm-20-2072. |
| 140 | Zhou M, Hua L, Wang YF, et al. Oral Huzhang granules for the treatment of acute gouty arthritis: protocol for a double-blind, randomized, controlled trial. Trials 2022; 23: 248. Journal article. DOI: 10.1186/s13063-022-06188-x. |
| 141 | Abdellatif AA and Elkhalili N. Management of gouty arthritis in patients with chronic kidney disease. American journal of therapeutics 2014; 21: 523-534. 2012/09/11. DOI: 10.1097/MJT.0b013e318250f83d. |
| 142 | Ahern MJ, Reid C, Gordon TP, et al. Does colchicine work? The results of the first controlled study in acute gout. Australian and New Zealand journal of medicine 1987; 17: 301-304. 1987/06/01. DOI: 10.1111/j.1445-5994.1987.tb01232.x. |
| 143 | Akgul O, Kilic E, Kilic G, et al. Efficacy and safety of biologic treatments in Familial Mediterranean Fever. The American journal of the medical sciences 2013; 346: 137-141. 2013/01/02. DOI: 10.1097/MAJ.0b013e318277083b. |
| 144 | Aran S, Malekzadeh S and Seifirad S. A double-blind randomized controlled trial appraising the symptom-modifying effects of colchicine on osteoarthritis of the knee. Clin Exp Rheumatol 2011; 29: 513-518. 2011/06/07. |
| 145 | Becker MA, Schumacher HR, Jr., Wortmann RL, et al. Febuxostat compared with allopurinol in patients with hyperuricemia and gout. The New England journal of medicine 2005; 353: 2450-2461. 2005/12/13. DOI: 10.1056/NEJMoa050373. |
| 146 | Becker MA, Schumacher HR, Jr., Wortmann RL, et al. Febuxostat, a novel nonpurine selective inhibitor of xanthine oxidase: a twenty-eight-day, multicenter, phase II, randomized, double-blind, placebo-controlled, dose-response clinical trial examining safety and efficacy in patients with gout. Arthritis Rheum 2005; 52: 916-923. 2005/03/08. DOI: 10.1002/art.20935. |
| 147 | Ben-Chetrit E, Peleg H, Aamar S, et al. The spectrum of MEFV clinical presentations--is it familial Mediterranean fever only? Rheumatology (Oxford, England) 2009; 48: 1455-1459. 2009/10/20. DOI: 10.1093/rheumatology/kep296. |
| 148 | BenEzra D, Cohen E, Chajek T, et al. Evaluation of conventional therapy versus cyclosporine A in Behçet's syndrome. Transplant Proc 1988; 20: 136-143. 1988/06/01. |
| 149 | Bluestone R, Kippen I and Klinenberg JR. Effect of drugs on urate binding to plasma proteins. British medical journal 1969; 4: 590-593. 1969/12/06. DOI: 10.1136/bmj.4.5683.590. |
| 150 | Boland EW. NONSPECIFIC ANTI-INFLAMMATORY AGENTS. SOME NOTES ON THEIR PRACTICAL APPLICATION, ESPECIALLY IN RHEUMATIC DISORDERS. California medicine 1964; 100: 145-155. 1964/03/01. |
| 151 | Borstad GC, Bryant LR, Abel MP, et al. Colchicine for prophylaxis of acute flares when initiating allopurinol for chronic gouty arthritis. The Journal of rheumatology 2004; 31: 2429-2432. 2004/12/01. |
| 152 | Bruce SP. Febuxostat: a selective xanthine oxidase inhibitor for the treatment of hyperuricemia and gout. The Annals of pharmacotherapy 2006; 40: 2187-2194. 2006/11/30. DOI: 10.1345/aph.1H121. |
| 153 | Calgüneri M, Kiraz S, Ertenli I, et al. The effect of prophylactic penicillin treatment on the course of arthritis episodes in patients with Behçet's disease. A randomized clinical trial. Arthritis Rheum 1996; 39: 2062-2065. 1996/12/01. DOI: 10.1002/art.1780391216. |
| 154 | Conaghan PG and Day RO. Risks and benefits of drugs used in the management and prevention of gout. Drug safety 1994; 11: 252-258. 1994/10/01. DOI: 10.2165/00002018-199411040-00004. |
| 155 | Cronstein BN and Terkeltaub R. The inflammatory process of gout and its treatment. Arthritis Res Ther 2006; 8 Suppl 1: S3. 2006/07/06. DOI: 10.1186/ar1908. |
| 156 | Curiel RV and Guzman NJ. Challenges associated with the management of gouty arthritis in patients with chronic kidney disease: a systematic review. Seminars in arthritis and rheumatism 2012; 42: 166-178. 2012/05/09. DOI: 10.1016/j.semarthrit.2012.03.013. |
| 157 | Das SK, Mishra K, Ramakrishnan S, et al. A randomized controlled trial to evaluate the slow-acting symptom modifying effects of a regimen containing colchicine in a subset of patients with osteoarthritis of the knee. Osteoarthritis Cartilage 2002; 10: 247-252. 2002/04/13. DOI: 10.1053/joca.2002.0516. |
| 158 | Das SK, Ramakrishnan S, Mishra K, et al. A randomized controlled trial to evaluate the slow-acting symptom-modifying effects of colchicine in osteoarthritis of the knee: a preliminary report. Arthritis Rheum 2002; 47: 280-284. 2002/07/13. DOI: 10.1002/art.10455. |
| 159 | Demiroglu H, Ozcebe OI, Barista I, et al. Interferon alfa-2b, colchicine, and benzathine penicillin versus colchicine and benzathine penicillin in Behçet's disease: a randomised trial. Lancet 2000; 355: 605-609. 2000/03/04. DOI: 10.1016/s0140-6736(99)05131-4. |
| 160 | Ernst ME and Fravel MA. Febuxostat: a selective xanthine-oxidase/xanthine-dehydrogenase inhibitor for the management of hyperuricemia in adults with gout. Clinical therapeutics 2009; 31: 2503-2518. 2010/01/30. DOI: 10.1016/j.clinthera.2009.11.033. |
| 161 | Genvo MF, Faure M and Thivolet J. [Treatment of aphthosis with thalidomide and with colchicine]. Dermatologica 1984; 168: 182-188. 1984/01/01. |
| 162 | Gertz MA and Kyle RA. Amyloidosis: prognosis and treatment. Seminars in arthritis and rheumatism 1994; 24: 124-138. 1994/10/01. DOI: 10.1016/s0049-0172(05)80006-x. |
| 163 | Groff GD, Franck WA and Raddatz DA. Systemic steroid therapy for acute gout: a clinical trial and review of the literature. Seminars in arthritis and rheumatism 1990; 19: 329-336. 1990/06/01. DOI: 10.1016/0049-0172(90)90070-v. |
| 164 | Huang X, Du H, Gu J, et al. An allopurinol-controlled, multicenter, randomized, double-blind, parallel between-group, comparative study of febuxostat in Chinese patients with gout and hyperuricemia. Int J Rheum Dis 2014; 17: 679-686. 2014/01/29. DOI: 10.1111/1756-185x.12266. |
| 165 | Janssens HJ, Janssen M, van de Lisdonk EH, et al. Use of oral prednisolone or naproxen for the treatment of gout arthritis: a double-blind, randomised equivalence trial. Lancet 2008; 371: 1854-1860. 2008/06/03. DOI: 10.1016/s0140-6736(08)60799-0. |
| 166 | Janssens HJ, Lucassen PL, Van de Laar FA, et al. Systemic corticosteroids for acute gout. The Cochrane database of systematic reviews 2008; 2008: Cd005521. 2008/04/22. DOI: 10.1002/14651858.CD005521.pub2. |
| 167 | Jones G, Crotty M and Brooks P. Psoriatic arthritis: a quantitative overview of therapeutic options. The Psoriatic Arthritis Meta-Analysis Study Group. British journal of rheumatology 1997; 36: 95-99. 1997/01/01. DOI: 10.1093/rheumatology/36.1.95. |
| 168 | Jones G, Crotty M and Brooks P. Interventions for psoriatic arthritis. The Cochrane database of systematic reviews 2000: Cd000212. 2000/07/25. DOI: 10.1002/14651858.Cd000212. |
| 169 | Jones G, Crotty M and Brooks P. Interventions for psoriatic arthritis. The Cochrane database of systematic reviews 2000; 2000: Cd000212. 2000/05/05. DOI: 10.1002/14651858.Cd000212. |
| 170 | Khanna PP, Gladue HS, Singh MK, et al. Treatment of acute gout: a systematic review. Seminars in arthritis and rheumatism 2014; 44: 31-38. 2014/03/22. DOI: 10.1016/j.semarthrit.2014.02.003. |
| 171 | Lanfranchi R and Volpi A. [Results of the clinical trial of intra-articular thiocolchicoside in scapulo-humeral periarthritis]. Minerva medica 1968; 59: 4678-4681. 1968/10/31. |
| 172 | Leong KH and Feng PH. Gout. Singapore medical journal 1992; 33: 393-394. 1992/08/01. |
| 173 | Leventhal LJ, Levin RW and Bomalaski JS. Peripheral arthrocentesis in the work-up of acute low back pain. Archives of physical medicine and rehabilitation 1990; 71: 253-254. 1990/03/01. |
| 174 | Matucci-Cerinic M, Ceruso M, Lotti T, et al. The medical and surgical treatment of finger clubbing and hypertrophic osteoarthropathy. A blind study with colchicine and a surgical approach to finger clubbing reduction. Clin Exp Rheumatol 1992; 10 Suppl 7: 67-70. 1992/05/01. |
| 175 | McKendry RJ, Kraag G, Seigel S, et al. Therapeutic value of colchicine in the treatment of patients with psoriatic arthritis. Ann Rheum Dis 1993; 52: 826-828. 1993/11/01. DOI: 10.1136/ard.52.11.826. |
| 176 | Moi JH, Sriranganathan MK, Edwards CJ, et al. Lifestyle interventions for acute gout. The Cochrane database of systematic reviews 2013; 2013: Cd010519. 2013/11/05. DOI: 10.1002/14651858.CD010519.pub2. |
| 177 | Navarra S, Rubin BR, Yu Q, et al. Association of baseline disease and patient characteristics with response to etoricoxib and indomethacin for acute gout. Curr Med Res Opin 2007; 23: 1685-1691. 2007/06/26. DOI: 10.1185/030079907x210750. |
| 178 | Nilsson F, Norberg B, Frederiksen B, et al. The role of the MA-sensitive leukocyte chemotaxis in rheumatoid arthritis. A randomized double-blind clinical trial of griseofulvin treatment. Scand J Rheumatol 1983; 12: 113-118. 1983/01/01. DOI: 10.3109/03009748309102895. |
| 179 | Nuki G. Colchicine: its mechanism of action and efficacy in crystal-induced inflammation. Current rheumatology reports 2008; 10: 218-227. 2008/07/22. DOI: 10.1007/s11926-008-0036-3. |
| 180 | Ozen S. New interest in an old disease: familial Mediterranean fever. Clin Exp Rheumatol 1999; 17: 745-749. 1999/12/28. |
| 181 | Ozen S. Familial mediterranean fever: revisiting an ancient disease. European journal of pediatrics 2003; 162: 449-454. 2003/05/17. DOI: 10.1007/s00431-003-1223-x. |
| 182 | Paulus HE, Schlosstein LH, Godfrey RG, et al. Prophylactic colchicine therapy of intercritical gout. A placebo-controlled study of probenecid-treated patients. Arthritis Rheum 1974; 17: 609-614. 1974/09/01. DOI: 10.1002/art.1780170517. |
| 183 | Richette P and Bardin T. Colchicine for the treatment of gout. Expert opinion on pharmacotherapy 2010; 11: 2933-2938. 2010/11/06. DOI: 10.1517/14656566.2010.529432. |
| 184 | Ryu HJ, Song R, Kim HW, et al. Clinical risk factors for adverse events in allopurinol users. Journal of clinical pharmacology 2013; 53: 211-216. 2013/02/26. DOI: 10.1177/0091270012439715. |
| 185 | Saenz A, Ausejo M, Shea B, et al. Pharmacotherapy for Behcet's syndrome. The Cochrane database of systematic reviews 2000; 1998: Cd001084. 2000/05/05. DOI: 10.1002/14651858.Cd001084. |
| 186 | Schlesinger N. Overview of the management of acute gout and the role of adrenocorticotropic hormone. Drugs 2008; 68: 407-415. 2008/03/06. DOI: 10.2165/00003495-200868040-00002. |
| 187 | Schlesinger N, Alten RE, Bardin T, et al. Canakinumab for acute gouty arthritis in patients with limited treatment options: results from two randomised, multicentre, active-controlled, double-blind trials and their initial extensions. Ann Rheum Dis 2012; 71: 1839-1848. 2012/05/16. DOI: 10.1136/annrheumdis-2011-200908. |
| 188 | Schlesinger N, De Meulemeester M, Pikhlak A, et al. Canakinumab relieves symptoms of acute flares and improves health-related quality of life in patients with difficult-to-treat Gouty Arthritis by suppressing inflammation: results of a randomized, dose-ranging study. Arthritis Res Ther 2011; 13: R53. 2011/03/29. DOI: 10.1186/ar3297. |
| 189 | Schlesinger N, Detry MA, Holland BK, et al. Local ice therapy during bouts of acute gouty arthritis. The Journal of rheumatology 2002; 29: 331-334. 2002/02/13. |
| 190 | Schlesinger N, Mysler E, Lin HY, et al. Canakinumab reduces the risk of acute gouty arthritis flares during initiation of allopurinol treatment: results of a double-blind, randomised study. Ann Rheum Dis 2011; 70: 1264-1271. 2011/05/05. DOI: 10.1136/ard.2010.144063. |
| 191 | Schlesinger N, Schumacher R, Catton M, et al. Colchicine for acute gout. The Cochrane database of systematic reviews 2006: Cd006190. 2006/10/21. DOI: 10.1002/14651858.Cd006190. |
| 192 | Scott JT. Comparison of allopurinol and probenecid. Ann Rheum Dis 1966; 25: 623-626. 1966/11/01. DOI: 10.1136/ard.25.Suppl_6.623. |
| 193 | Seideman P, Fjellner B and Johannesson A. Psoriatic arthritis treated with oral colchicine. The Journal of rheumatology 1987; 14: 777-779. 1987/08/01. |
| 194 | Sharma A, Provenzale D, McKusick A, et al. Interstitial pneumonitis after low-dose methotrexate therapy in primary biliary cirrhosis. Gastroenterology 1994; 107: 266-270. 1994/07/01. DOI: 10.1016/0016-5085(94)90085-x. |
| 195 | Siegel LB, Alloway JA and Nashel DJ. Comparison of adrenocorticotropic hormone and triamcinolone acetonide in the treatment of acute gouty arthritis. The Journal of rheumatology 1994; 21: 1325-1327. 1994/07/01. |
| 196 | So A, De Meulemeester M, Pikhlak A, et al. Canakinumab for the treatment of acute flares in difficult-to-treat gouty arthritis: Results of a multicenter, phase II, dose-ranging study. Arthritis Rheum 2010; 62: 3064-3076. 2010/06/10. DOI: 10.1002/art.27600. |
| 197 | Stack J, Ryan J and McCarthy G. Colchicine: New Insights to an Old Drug. American journal of therapeutics 2015; 22: e151-157. 2013/10/09. DOI: 10.1097/01.mjt.0000433937.07244.e1. |
| 198 | Sutaria S, Katbamna R and Underwood M. Effectiveness of interventions for the treatment of acute and prevention of recurrent gout--a systematic review. Rheumatology (Oxford, England) 2006; 45: 1422-1431. 2006/04/25. DOI: 10.1093/rheumatology/kel071. |
| 199 | Talbott JH. Diagnosis and treatment of gouty arthritis. California medicine 1953; 79: 220-226. 1953/09/01. |
| 200 | Taylor TH, Mecchella JN, Larson RJ, et al. Initiation of allopurinol at first medical contact for acute attacks of gout: a randomized clinical trial. The American journal of medicine 2012; 125: 1126-1134.e1127. 2012/10/27. DOI: 10.1016/j.amjmed.2012.05.025. |
| 201 | Ter Haar N, Lachmann H, Özen S, et al. Treatment of autoinflammatory diseases: results from the Eurofever Registry and a literature review. Ann Rheum Dis 2013; 72: 678-685. 2012/07/04. DOI: 10.1136/annrheumdis-2011-201268. |
| 202 | Terkeltaub RA, Furst DE, Bennett K, et al. High versus low dosing of oral colchicine for early acute gout flare: Twenty-four-hour outcome of the first multicenter, randomized, double-blind, placebo-controlled, parallel-group, dose-comparison colchicine study. Arthritis Rheum 2010; 62: 1060-1068. 2010/02/05. DOI: 10.1002/art.27327. |
| 203 | Tetik O, Doral MN, Atay AO, et al. Influence of irrigation solutions combined with colchicine and diclofenac sodium on articular cartilage in a rat model. Knee surgery, sports traumatology, arthroscopy : official journal of the ESSKA 2004; 12: 503-509. 2004/04/07. DOI: 10.1007/s00167-003-0470-8. |
| 204 | van Echteld I, Wechalekar MD, Schlesinger N, et al. Colchicine for acute gout. The Cochrane database of systematic reviews 2014: Cd006190. 2014/08/16. DOI: 10.1002/14651858.CD006190.pub2. |
| 205 | Verne GN, Eaker EY, Davis RH, et al. Colchicine is an effective treatment for patients with chronic constipation: an open-label trial. Digestive diseases and sciences 1997; 42: 1959-1963. 1997/10/23. DOI: 10.1023/a:1018883731556. |
| 206 | Vogel Y, Büchner NJ, Haverkamp T, et al. [Familial Mediterranean fever. Rare manifestation without fever and with inconspicuous family case history]. Deutsche medizinische Wochenschrift (1946) 2008; 133: 1621-1624. 2008/07/25. DOI: 10.1055/s-0028-1082777. |
| 207 | Wallace SI, Bernstein D and Diamond H. Diagnostic value of the colchicine therapeutic trial. Jama 1967; 199: 525-528. 1967/02/20. |
| 208 | Wang Y, Wang L, Li E, et al. Chuanhu anti-gout mixture versus colchicine for acute gouty arthritis: a randomized, double-blind, double-dummy, non-inferiority trial. Int J Med Sci 2014; 11: 880-885. 2014/07/12. DOI: 10.7150/ijms.9165. |
| 209 | Wechalekar MD, Vinik O, Moi JH, et al. The efficacy and safety of treatments for acute gout: results from a series of systematic literature reviews including Cochrane reviews on intraarticular glucocorticoids, colchicine, nonsteroidal antiinflammatory drugs, and interleukin-1 inhibitors. The Journal of rheumatology Supplement 2014; 92: 15-25. 2014/09/03. DOI: 10.3899/jrheum.140458. |
| 210 | Wortmann RL, Macdonald PA, Hunt B, et al. Effect of prophylaxis on gout flares after the initiation of urate-lowering therapy: analysis of data from three phase III trials. Clinical therapeutics 2010; 32: 2386-2397. 2011/03/01. DOI: 10.1016/j.clinthera.2011.01.008. |
| 211 | Yang LP. Oral colchicine (Colcrys): in the treatment and prophylaxis of gout. Drugs 2010; 70: 1603-1613. 2010/08/07. DOI: 10.2165/11205470-000000000-00000. |
| 212 | Yurdakul S, Mat C, Tüzün Y, et al. A double-blind trial of colchicine in Behçet's syndrome. Arthritis Rheum 2001; 44: 2686-2692. 2001/11/17. DOI: 10.1002/1529-0131(200111)44:11<2686::aid-art448>3.0.co;2-h. |
| 213 | Amarilyo G, Rothman D, Manthiram K, et al. Consensus treatment plans for periodic fever, aphthous stomatitis, pharyngitis and adenitis syndrome (PFAPA): a framework to evaluate treatment responses from the childhood arthritis and rheumatology research alliance (CARRA) PFAPA work group. Pediatr Rheumatol Online J 2020; 18: 31. 2020/04/16. DOI: 10.1186/s12969-020-00424-x. |
| 214 | Aviel YB, Rawan S, Fahoum S, et al. Discontinuation of Colchicine Therapy in Children With Familial Mediterranean Fever. The Journal of rheumatology 2021; 48: 1732-1735. 2021/05/17. DOI: 10.3899/jrheum.201158. |
| 215 | Banerjee M, Pal R, Maisnam I, et al. Serum uric acid lowering and effects of sodium-glucose cotransporter-2 inhibitors on gout: A meta-analysis and meta-regression of randomized controlled trials. Diabetes, obesity & metabolism 2023; 25: 2697-2703. 2023/06/19. DOI: 10.1111/dom.15157. |
| 216 | Barry A, Helget LN, Androsenko M, et al. Comparison of Gout Flares With the Initiation of Treat-to-Target Allopurinol and Febuxostat: A Post-Hoc Analysis of a Randomized Multicenter Trial. Arthritis & rheumatology (Hoboken, NJ) 2024 2024/06/27. DOI: 10.1002/art.42927. |
| 217 | Butt JH, Docherty KF, Claggett BL, et al. Association of Dapagliflozin Use With Clinical Outcomes and the Introduction of Uric Acid-Lowering Therapy and Colchicine in Patients With Heart Failure With and Without Gout: A Patient-Level Pooled Meta-analysis of DAPA-HF and DELIVER. JAMA cardiology 2023; 8: 386-393. 2023/02/23. DOI: 10.1001/jamacardio.2022.5608. |
| 218 | Cheng Y, Huang X, Tang Y, et al. Effects of evodiamine on ROS/TXNIP/NLRP3 pathway against gouty arthritis. Naunyn-Schmiedeberg's archives of pharmacology 2024; 397: 1015-1023. 2023/08/09. DOI: 10.1007/s00210-023-02654-8. |
| 219 | Chuang JP, Lee JC, Leu TH, et al. Association of gout and colorectal cancer in Taiwan: a nationwide population-based cohort study. BMJ open 2019; 9: e028892. 2019/10/12. DOI: 10.1136/bmjopen-2019-028892. |
| 220 | Conley B, Bunzli S, Bullen J, et al. What are the core recommendations for gout management in first line and specialist care? Systematic review of clinical practice guidelines. BMC rheumatology 2023; 7: 15. 2023/06/15. DOI: 10.1186/s41927-023-00335-w. |
| 221 | Dalbeth N, Schumacher HR, Fransen J, et al. Survey Definitions of Gout for Epidemiologic Studies: Comparison With Crystal Identification as the Gold Standard. Arthritis care & research 2016; 68: 1894-1898. 2016/10/30. DOI: 10.1002/acr.22896. |
| 222 | Davis CR, Ruediger CD, Dyer KA, et al. Colchicine is not effective for reducing osteoarthritic hand pain compared to placebo: a randomised, placebo-controlled trial (COLAH). Osteoarthritis Cartilage 2021; 29: 208-214. 2020/11/25. DOI: 10.1016/j.joca.2020.11.002. |
| 223 | Estiverne C and Mount DB. The Management of Gout in Renal Disease. Seminars in nephrology 2020; 40: 600-613. 2021/03/09. DOI: 10.1016/j.semnephrol.2020.12.007. |
| 224 | Finckh A, Mc Carthy GM, Madigan A, et al. Methotrexate in chronic-recurrent calcium pyrophosphate deposition disease: no significant effect in a randomized crossover trial. Arthritis Res Ther 2014; 16: 458. 2014/10/16. DOI: 10.1186/s13075-014-0458-4. |
| 225 | Fiolet ATL, Nidorf SM, Mosterd A, et al. Colchicine in Stable Coronary Artery Disease. Clinical therapeutics 2019; 41: 30-40. 2018/11/07. DOI: 10.1016/j.clinthera.2018.09.011. |
| 226 | Giannopoulos G and Deftereos S. Effects of colchicine on risk of cardiovascular events among patients with gout: as evidence accrues, is it time for a randomised trial? Ann Rheum Dis 2016; 75: e28. 2016/02/20. DOI: 10.1136/annrheumdis-2016-209294. |
| 227 | Grygiel-Górniak B, Masiero E, Nevaneeth BC, et al. Rheumatic Diseases in Reproductive Age-the Possibilities and the Risks. Reproductive sciences (Thousand Oaks, Calif) 2023; 30: 111-123. 2022/04/02. DOI: 10.1007/s43032-022-00901-6. |
| 228 | Guma M, Dadpey B, Coras R, et al. Xanthine oxidase inhibitor urate-lowering therapy titration to target decreases serum free fatty acids in gout and suppresses lipolysis by adipocytes. Arthritis Res Ther 2022; 24: 175. 2022/07/26. DOI: 10.1186/s13075-022-02852-4. |
| 229 | Hashkes PJ and Huang B. The familial Mediterranean fever (FMF) 50 score: does it work in a controlled clinical trial? Re-analysis of the trial of rilonacept for patients with colchicine-resistant or intolerant FMF. The Israel Medical Association journal : IMAJ 2015; 17: 137-140. 2015/05/08. |
| 230 | He Y, Dai C, Shen J, et al. Effect of Baihu and Guizhi decoction in acute gouty arthritis: study protocol for a randomized controlled trial. Trials 2022; 23: 317. 2022/04/17. DOI: 10.1186/s13063-022-06194-z. |
| 231 | Hegaze AH, Hamdi AS, Alqrache AT, et al. Unusual Case of Gouty Arthritis of the Second Distal Interphalangeal Joint (Second Toe). Cureus 2020; 12: e11405. 2020/12/15. DOI: 10.7759/cureus.11405. |
| 232 | Heijman MWJ, Fiolet ATL, Mosterd A, et al. Association of Low-Dose Colchicine With Incidence of Knee and Hip Replacements : Exploratory Analyses From a Randomized, Controlled, Double-Blind Trial. Annals of internal medicine 2023; 176: 737-742. 2023/05/29. DOI: 10.7326/m23-0289. |
| 233 | Hill EM, Sky K, Sit M, et al. Does starting allopurinol prolong acute treated gout? A randomized clinical trial. Journal of clinical rheumatology : practical reports on rheumatic & musculoskeletal diseases 2015; 21: 120-125. 2015/03/26. DOI: 10.1097/rhu.0000000000000235. |
| 234 | Janssen CA, Oude Voshaar MAH, Ten Klooster PM, et al. Development and validation of a patient-reported gout attack intensity score for use in gout clinical studies. Rheumatology (Oxford, England) 2019; 58: 1928-1934. 2019/03/13. DOI: 10.1093/rheumatology/kez064. |
| 235 | Jia E, Hu S, Geng H, et al. Zhengqing fengtongning sustained-release tablets prevents gout flares in the process of ULT: A randomized, positive control, double-blind, double-simulation, multicenter trial. Medicine (Baltimore) 2022; 101: e29199. 2022/05/14. DOI: 10.1097/md.0000000000029199. |
| 236 | Juraschek SP, Appel LJ and Miller ER, 3rd. Metoprolol Increases Uric Acid and Risk of Gout in African Americans With Chronic Kidney Disease Attributed to Hypertension. American journal of hypertension 2017; 30: 871-875. 2017/08/24. DOI: 10.1093/ajh/hpx113. |
| 237 | Kankam M, Hall J, Gillen M, et al. Pharmacokinetics, Pharmacodynamics, and Tolerability of Concomitant Multiple Dose Administration of Verinurad (RDEA3170) and Allopurinol in Adult Male Subjects With Gout. Journal of clinical pharmacology 2018; 58: 1214-1222. 2018/05/08. DOI: 10.1002/jcph.1119. |
| 238 | Kaul S, Gupta M, Bandyopadhyay D, et al. Gout Pharmacotherapy in Cardiovascular Diseases: A Review of Utility and Outcomes. American journal of cardiovascular drugs : drugs, devices, and other interventions 2021; 21: 499-512. 2020/12/29. DOI: 10.1007/s40256-020-00459-1. |
| 239 | Kesselheim AS, Franklin JM, Kim SC, et al. Reductions in Use of Colchicine after FDA Enforcement of Market Exclusivity in a Commercially Insured Population. Journal of general internal medicine 2015; 30: 1633-1638. 2015/04/10. DOI: 10.1007/s11606-015-3285-7. |
| 240 | Laosuksri P, Phrintrakul N, Gumtorntip W, et al. Non-loading versus loading low-dose colchicine in acute crystal-associated arthritis: A double-blinded randomized controlled study. Int J Rheum Dis 2023; 26: 2478-2488. 2023/10/20. DOI: 10.1111/1756-185x.14943. |
| 241 | Leung YY, Haaland B, Huebner JL, et al. Colchicine lack of effectiveness in symptom and inflammation modification in knee osteoarthritis (COLKOA): a randomized controlled trial. Osteoarthritis Cartilage 2018; 26: 631-640. 2018/02/10. DOI: 10.1016/j.joca.2018.01.026. |
| 242 | Leung YY, Huebner JL, Haaland B, et al. Synovial fluid pro-inflammatory profile differs according to the characteristics of knee pain. Osteoarthritis Cartilage 2017; 25: 1420-1427. 2017/04/24. DOI: 10.1016/j.joca.2017.04.001. |
| 243 | Leung YY, Thumboo J, Wong BS, et al. Colchicine effectiveness in symptom and inflammation modification in knee osteoarthritis (COLKOA): study protocol for a randomized controlled trial. Trials 2015; 16: 200. 2015/05/01. DOI: 10.1186/s13063-015-0726-x. |
| 244 | Liang H, Wu Y, Zhang W, et al. Efficacy and Safety of Acupuncture Combined with Herbal Medicine in Treating Gouty Arthritis: Meta-Analysis of Randomized Controlled Trials. Evid Based Complement Alternat Med 2021; 2021: 8161731. 2022/01/11. DOI: 10.1155/2021/8161731. |
| 245 | Lin N, Dai Q, Zhang Y, et al. Chinese classical decoction Wuwei Xiaodu Drink alleviates gout arthritis by suppressing NLRP3-Mediated inflammation. Front Pharmacol 2024; 15: 1388753. 2024/08/12. DOI: 10.3389/fphar.2024.1388753. |
| 246 | Liu P, Chen Y, Wang B, et al. Expression of microRNAs in the plasma of patients with acute gouty arthritis and the effects of colchicine and etoricoxib on the differential expression of microRNAs. Archives of medical science : AMS 2019; 15: 1047-1055. 2019/07/31. DOI: 10.5114/aoms.2018.75502. |
| 247 | Liu W, Wang H, Su C, et al. The Evaluation of the Efficacy and Safety of Oral Colchicine in the Treatment of Knee Osteoarthritis: A Meta-Analysis of Randomized Controlled Trails. BioMed research international 2022; 2022: 2381828. 2022/02/09. DOI: 10.1155/2022/2381828. |
| 248 | Liu X, Sun D, Ma X, et al. Benefit-risk of corticosteroids in acute gout patients: An updated meta-analysis and economic evaluation. Steroids 2017; 128: 89-94. 2017/09/14. DOI: 10.1016/j.steroids.2017.09.002. |
| 249 | Lu H, Liu W, Fan Y, et al. Efficacy and Safety of Qinpi Tongfeng Formula Combined with Bloodletting Therapy in the Treatment of Acute Gouty Arthritis: A Study Protocol for a Randomized Controlled Trial. Evid Based Complement Alternat Med 2022; 2022: 3147319. 2022/02/01. DOI: 10.1155/2022/3147319. |
| 250 | Maher D, Reeve E, Hopkins A, et al. Comparative Risk of Gout Flares When Initiating or Escalating Various Urate-Lowering Therapy: A Systematic Review With Network Meta-Analysis. Arthritis care & research 2024; 76: 871-881. 2024/02/02. DOI: 10.1002/acr.25309. |
| 251 | Mandell BF, Fields TR, Edwards NL, et al. Post-hoc analysis of pegloticase pivotal trials in chronic refractory gout: relationship between fluctuations in plasma urate levels and acute flares. Clin Exp Rheumatol 2021; 39: 1085-1092. 2021/01/12. DOI: 10.55563/clinexprheumatol/b7jjnb. |
| 252 | Mansouri N, Marjani M, Tabarsi P, et al. Successful Treatment of Covid-19 Associated Cytokine Release Syndrome with Colchicine. A Case Report and Review of Literature. Immunological investigations 2021; 50: 884-890. 2020/07/08. DOI: 10.1080/08820139.2020.1789655. |
| 253 | McKenzie BJ, Wechalekar MD, Johnston RV, et al. Colchicine for acute gout. The Cochrane database of systematic reviews 2021; 8: Cd006190. 2021/08/27. DOI: 10.1002/14651858.CD006190.pub3. |
| 254 | Moi JH, Sriranganathan MK, Falzon L, et al. Lifestyle interventions for the treatment of gout: a summary of 2 Cochrane systematic reviews. The Journal of rheumatology Supplement 2014; 92: 26-32. 2014/09/03. DOI: 10.3899/jrheum.140459. |
| 255 | Naifa G, Totikidis G, Alexiadou S, et al. Intravenous γ Globulin for Intractable Abdominal Pain due to IgA Vasculitis. Case reports in pediatrics 2020; 2020: 8867621. 2020/10/31. DOI: 10.1155/2020/8867621. |
| 256 | Narang RK and Dalbeth N. Management of complex gout in clinical practice: Update on therapeutic approaches. Best practice & research Clinical rheumatology 2018; 32: 813-834. 2019/08/21. DOI: 10.1016/j.berh.2019.03.010. |
| 257 | Nidorf SM. Seeing Colchicine in a New Light: Repurposing Low-dose Colchicine for Secondary Prevention of Cardiovascular Disease. Clinical therapeutics 2023; 45: 1029-1033. 2023/07/30. DOI: 10.1016/j.clinthera.2023.07.007. |
| 258 | Nidorf SM and Thompson PL. Why Colchicine Should Be Considered for Secondary Prevention of Atherosclerosis: An Overview. Clinical therapeutics 2019; 41: 41-48. 2018/12/29. DOI: 10.1016/j.clinthera.2018.11.016. |
| 259 | Oh YJ, Lee YJ, Lee E, et al. Cancer risk in Korean patients with gout. The Korean journal of internal medicine 2022; 37: 460-467. 2020/09/03. DOI: 10.3904/kjim.2020.259. |
| 260 | Oliviero F, Galozzi P, Scanu A, et al. Polydatin Prevents Calcium Pyrophosphate Crystal-Induced Arthritis in Mice. Nutrients 2021; 13 2021/04/04. DOI: 10.3390/nu13030929. |
| 261 | Parisa N, Hidayat R, Maritska Z, et al. Evaluation of the anti-gout effect of Sonchus Arvensis on monosodium urate crystal-induced gout arthritis via anti-inflammatory action - an in vivo study. Medicine and pharmacy reports 2021; 94: 358-365. 2021/08/26. DOI: 10.15386/mpr-1959. |
| 262 | Parperis K. Open-label randomised pragmatic trial (CONTACT) comparing naproxen and low-dose colchicine for the treatment of gout flares in primary care. Ann Rheum Dis 2021; 80: e202. 2019/11/30. DOI: 10.1136/annrheumdis-2019-216643. |
| 263 | Parperis K, Papachristodoulou E, Kakoullis L, et al. Management of calcium pyrophosphate crystal deposition disease: A systematic review. Seminars in arthritis and rheumatism 2021; 51: 84-94. 2020/12/29. DOI: 10.1016/j.semarthrit.2020.10.005. |
| 264 | Pascart T, Lancrenon S, Lanz S, et al. GOSPEL 2 - Colchicine for the treatment of gout flares in France - a GOSPEL survey subgroup analysis. Doses used in common practices regardless of renal impairment and age. Joint bone spine 2016; 83: 687-693. 2016/02/28. DOI: 10.1016/j.jbspin.2015.10.006. |
| 265 | Pascart T and Richette P. Colchicine in Gout: An Update. Current pharmaceutical design 2018; 24: 684-689. 2018/01/18. DOI: 10.2174/1381612824999180115103951. |
| 266 | Pascart T, Robinet P, Ottaviani S, et al. Evaluating the safety and short-term equivalence of colchicine versus prednisone in older patients with acute calcium pyrophosphate crystal arthritis (COLCHICORT): an open-label, multicentre, randomised trial. Lancet Rheumatol 2023; 5: e523-e531. 2024/01/22. DOI: 10.1016/s2665-9913(23)00165-0. |
| 267 | Pascual E, Andrés M and Sivera F. Methotrexate: should it still be considered for chronic calcium pyrophosphate crystal disease? Arthritis Res Ther 2015; 17: 89. 2015/04/18. DOI: 10.1186/s13075-015-0598-1. |
| 268 | Perez-Ruiz F, Sundy JS, Miner JN, et al. Lesinurad in combination with allopurinol: results of a phase 2, randomised, double-blind study in patients with gout with an inadequate response to allopurinol. Ann Rheum Dis 2016; 75: 1074-1080. 2016/01/09. DOI: 10.1136/annrheumdis-2015-207919. |
| 269 | Pisaniello HL, Fisher MC, Farquhar H, et al. Efficacy and safety of gout flare prophylaxis and therapy use in people with chronic kidney disease: a Gout, Hyperuricemia and Crystal-Associated Disease Network (G-CAN)-initiated literature review. Arthritis Res Ther 2021; 23: 130. 2021/04/30. DOI: 10.1186/s13075-021-02416-y. |
| 270 | Poiley J, Steinberg AS, Choi YJ, et al. A Randomized, Double-Blind, Active- and Placebo-Controlled Efficacy and Safety Study of Arhalofenate for Reducing Flare in Patients With Gout. Arthritis & rheumatology (Hoboken, NJ) 2016; 68: 2027-2034. 2016/03/19. DOI: 10.1002/art.39684. |
| 271 | Pu MJ, Yao CJ, Liu LM, et al. Traditional Chinese medicine for gouty arthritis: A protocol for meta-analysis. Medicine (Baltimore) 2021; 100: e23699. 2021/02/07. DOI: 10.1097/md.0000000000023699. |
| 272 | Qaseem A, Harris RP, Forciea MA, et al. Management of Acute and Recurrent Gout: A Clinical Practice Guideline From the American College of Physicians. Annals of internal medicine 2017; 166: 58-68. 2016/11/02. DOI: 10.7326/m16-0570. |
| 273 | Qian X, Jiang Y, Luo Y, et al. The Anti-hyperuricemia and Anti-inflammatory Effects of Atractylodes Macrocephala in Hyperuricemia and Gouty Arthritis Rat Models. Combinatorial chemistry & high throughput screening 2023; 26: 950-964. 2022/06/07. DOI: 10.2174/1386207325666220603101540. |
| 274 | Qiao TX, Tang CL, Qiu L, et al. [Cathepsin-B involved in effect of electroacupuncture by inhibiting the activation of NLRP3 inflammasome in rats with acute gouty arthritis]. Zhen ci yan jiu = Acupuncture research 2021; 46: 295-300. 2021/05/02. DOI: 10.13702/j.1000-0607.200240. |
| 275 | Qiu L, Tang CL, Huang SQ, et al. [Effect of Electroacupuncture on Synovial M 1/M 2 Macrophage Polarization in Rats with Acute Gouty Arthritis]. Zhen ci yan jiu = Acupuncture research 2018; 43: 767-772. 2018/12/27. DOI: 10.13702/j.1000-0607.180498. |
| 276 | Richter A, Truthmann J, Hummers E, et al. Prednisolone Versus Colchicine for Acute Gout in Primary Care: statistical analysis plan for the pragmatic, multicenter, randomized, and double-blinded COPAGO non-inferiority trial. Trials 2024; 25: 229. 2024/04/04. DOI: 10.1186/s13063-024-08066-0. |
| 277 | Roddy E, Clarkson K, Blagojevic-Bucknall M, et al. Open-label randomised pragmatic trial (CONTACT) comparing naproxen and low-dose colchicine for the treatment of gout flares in primary care. Ann Rheum Dis 2020; 79: 276-284. 2019/11/02. DOI: 10.1136/annrheumdis-2019-216154. |
| 278 | Roddy E and Mallen CD. Response to: 'Open-label randomised pragmatic trial (CONTACT) comparing naproxen and low-dose colchicine for the treatment of gout flares in primary care' by Parperis et al. Ann Rheum Dis 2021; 80: e203. 2019/12/06. DOI: 10.1136/annrheumdis-2019-216671. |
| 279 | Saag KG, Becker MA, Whelton A, et al. Efficacy and Safety of Febuxostat Extended and Immediate Release in Patients With Gout and Renal Impairment: A Phase III Placebo-Controlled Study. Arthritis & rheumatology (Hoboken, NJ) 2019; 71: 143-153. 2018/08/04. DOI: 10.1002/art.40685. |
| 280 | Saag KG, Khanna PP, Keenan RT, et al. A Randomized, Phase II Study Evaluating the Efficacy and Safety of Anakinra in the Treatment of Gout Flares. Arthritis & rheumatology (Hoboken, NJ) 2021; 73: 1533-1542. 2021/02/20. DOI: 10.1002/art.41699. |
| 281 | Sandhu T, Tieng A, Chilimuri S, et al. A Case Control Study to Evaluate the Impact of Colchicine on Patients Admitted to the Hospital with Moderate to Severe COVID-19 Infection. Can J Infect Dis Med Microbiol 2020; 2020: 8865954. 2020/11/03. DOI: 10.1155/2020/8865954. |
| 282 | Sattui SE, Crow MK and Navarro-Millán I. The role of immunomodulatory medications in the treatment of COVID-19. Current opinion in rheumatology 2021; 33: 431-445. 2021/08/17. DOI: 10.1097/bor.0000000000000817. |
| 283 | Seth R, Kydd AS, Buchbinder R, et al. Allopurinol for chronic gout. The Cochrane database of systematic reviews 2014; 2014: Cd006077. 2014/10/15. DOI: 10.1002/14651858.CD006077.pub3. |
| 284 | Seth R, Kydd AS, Falzon L, et al. Preventing attacks of acute gout when introducing urate-lowering therapy: a systematic literature review. The Journal of rheumatology Supplement 2014; 92: 42-47. 2014/09/03. DOI: 10.3899/jrheum.140461. |
| 285 | Shekelle PG, Newberry SJ, FitzGerald JD, et al. Management of Gout: A Systematic Review in Support of an American College of Physicians Clinical Practice Guideline. Annals of internal medicine 2017; 166: 37-51. 2016/11/02. DOI: 10.7326/m16-0461. |
| 286 | Shen R, Ma L and Zheng Y. Anti-inflammatory effects of luteolin on acute gouty arthritis rats via TLR/MyD88/NF-κB pathway. Zhong nan da xue xue bao Yi xue ban = Journal of Central South University Medical sciences 2020; 45: 115-122. 2020/05/10. DOI: 10.11817/j.issn.1672-7347.2020.190566. |
| 287 | Singh A, Molina-Garcia P, Hussain S, et al. Efficacy and safety of colchicine for the treatment of osteoarthritis: a systematic review and meta-analysis of intervention trials. Clinical rheumatology 2023; 42: 889-902. 2022/10/13. DOI: 10.1007/s10067-022-06402-w. |
| 288 | Solomon DH and Kim SC. Response to: 'Effects of colchicine on risk of cardiovascular events among patients with gout: as evidence accrues, is it time for a randomized trial?' by Giannopoulos and Deftereos. Ann Rheum Dis 2016; 75: e29. 2016/03/02. DOI: 10.1136/annrheumdis-2016-209316. |
| 289 | Stamp L, Horne A, Mihov B, et al. Is colchicine prophylaxis required with start-low go-slow allopurinol dose escalation in gout? A non-inferiority randomised double-blind placebo-controlled trial. Ann Rheum Dis 2023; 82: 1626-1634. 2023/09/01. DOI: 10.1136/ard-2023-224731. |
| 290 | Tang H, Xu G, Zheng Q, et al. Treatment for acute flares of gout: A protocol for systematic review. Medicine (Baltimore) 2020; 99: e19668. 2020/04/04. DOI: 10.1097/md.0000000000019668. |
| 291 | Terkeltaub R, Lee J, Min J, et al. Serum Urate-Lowering Efficacy and Safety of Tigulixostat in Gout Patients With Hyperuricemia: A Randomized, Double-Blind, Placebo-Controlled, Dose-Finding Trial. Arthritis & rheumatology (Hoboken, NJ) 2023; 75: 1275-1284. 2023/01/18. DOI: 10.1002/art.42447. |
| 292 | Truthmann J, Freyer Martins Pereira J, Richter A, et al. Prednisolone Versus Colchicine for Acute Gout in Primary Care (COPAGO): protocol for a two-arm multicentre, pragmatic, prospective, randomized, double-blind, controlled clinical trial of prednisolone and colchicine for non-inferiority with a parallel group design. Trials 2023; 24: 643. 2023/10/06. DOI: 10.1186/s13063-023-07666-6. |
| 293 | Uhlig T, Karoliussen LF, Sexton J, et al. One- and 2-year flare rates after treat-to-target and tight-control therapy of gout: results from the NOR-Gout study. Arthritis Res Ther 2022; 24: 88. 2022/04/22. DOI: 10.1186/s13075-022-02772-3. |
| 294 | van de Laar CJ, Janssen CA, Janssen M, et al. Model-based cost-effectiveness analyses comparing combinations of urate lowering therapy and anti-inflammatory treatment in gout patients. PloS one 2022; 17: e0261940. 2022/01/29. DOI: 10.1371/journal.pone.0261940. |
| 295 | van Durme CM, Wechalekar MD, Landewé RB, et al. Non-steroidal anti-inflammatory drugs for acute gout. The Cochrane database of systematic reviews 2021; 12: Cd010120. 2021/12/10. DOI: 10.1002/14651858.CD010120.pub3. |
| 296 | Vargas-Santos AB, Castelar-Pinheiro Gda R, Coutinho ES, et al. Adherence to the 2012 American College of Rheumatology (ACR) Guidelines for Management of Gout: A Survey of Brazilian Rheumatologists. PloS one 2015; 10: e0135805. 2015/08/15. DOI: 10.1371/journal.pone.0135805. |
| 297 | Voulgari PV, Venetsanopoulou AI and Drosos AA. Recent advances in the therapeutic management of calcium pyrophosphate deposition disease. Frontiers in medicine 2024; 11: 1327715. 2024/03/26. DOI: 10.3389/fmed.2024.1327715. |
| 298 | Waller A and Jordan KM. Use of febuxostat in the management of gout in the United Kingdom. Therapeutic advances in musculoskeletal disease 2017; 9: 55-64. 2017/03/04. DOI: 10.1177/1759720x16682010. |
| 299 | Wang GH, Zuo T, Li R, et al. [Effect of rebamipide on the acute gouty arthritis in rats induced by monosodium urate crystals]. Beijing da xue xue bao Yi xue ban = Journal of Peking University Health sciences 2021; 53: 716-720. 2021/08/17. DOI: 10.19723/j.issn.1671-167X.2021.04.016. |
| 300 | Wang H, Chen ST, Ding XJ, et al. Efficacy and safety of Huzhang Granule, a compound Chinese herbal medicine, for acute gouty arthritis: A double-blind, randomized controlled trial. J Integr Med 2024; 22: 270-278. 2024/03/30. DOI: 10.1016/j.joim.2024.03.008. |
| 301 | Wang P, Ren D, Chen Y, et al. Effect of sodium alginate addition to resveratrol on acute gouty arthritis. Cellular physiology and biochemistry : international journal of experimental cellular physiology, biochemistry, and pharmacology 2015; 36: 201-207. 2015/05/15. DOI: 10.1159/000374064. |
| 302 | Yamanaka H, Tamaki S, Ide Y, et al. Stepwise dose increase of febuxostat is comparable with colchicine prophylaxis for the prevention of gout flares during the initial phase of urate-lowering therapy: results from FORTUNE-1, a prospective, multicentre randomised study. Ann Rheum Dis 2018; 77: 270-276. 2017/11/06. DOI: 10.1136/annrheumdis-2017-211574. |
| 303 | Yang DH, Chen HC and Wei JC. Early urate-lowering therapy in gouty arthritis with acute flares: a double-blind placebo controlled clinical trial. Eur J Med Res 2023; 28: 10. 2023/01/08. DOI: 10.1186/s40001-022-00982-8. |
| 304 | Yoshida K, Choi HK and Solomon DH. Medications for gout and its comorbidities: mutual benefits? Current opinion in rheumatology 2021; 33: 145-154. 2021/01/06. DOI: 10.1097/bor.0000000000000784. |
| 305 | Yu CL, Lu F, Yu DH, et al. [Mechanism of acteoside in prevention and treatment of gouty arthritis based on liver metabolomics]. Zhongguo Zhong yao za zhi = Zhongguo zhongyao zazhi = China journal of Chinese materia medica 2024; 49: 224-231. 2024/02/26. DOI: 10.19540/j.cnki.cjcmm.20230808.402. |
| 306 | Yu J, Li L, Liu J, et al. Influence of intervention treatment by "heat-clearing and diuresis-promoting" prescription on NALP3, an inflammatory factor in acute gouty arthritis. J Orthop Surg Res 2022; 17: 162. 2022/03/17. DOI: 10.1186/s13018-022-03046-z. |
| 307 | Zhang CN, Huang XK, Luo Y, et al. [The effects of electro-acupuncture on the signaling pathway of TLR/MYD88 in ankle joint synovial tissue of acute gouty arthritis rats]. Sichuan da xue xue bao Yi xue ban = Journal of Sichuan University Medical science edition 2014; 45: 924-927. 2015/01/13. |
| 308 | Zhang CN, Huang XK, Luo Y, et al. [Effects of electro-acupuncture on expression of triggering receptor expressed on myeloid cells 1 in ankle joint synovial tissue of acute gouty arthritis rats]. Nan fang yi ke da xue xue bao = Journal of Southern Medical University 2015; 35: 133-136. 2015/01/24. |
| 309 | Zhang J, Sun W, Gao F, et al. Changes of serum uric acid level during acute gout flare and related factors. Frontiers in endocrinology 2023; 14: 1077059. 2023/03/11. DOI: 10.3389/fendo.2023.1077059. |
| 310 | Zhang M, Zhang Y, Terkeltaub R, et al. Effect of Dietary and Supplemental Omega-3 Polyunsaturated Fatty Acids on Risk of Recurrent Gout Flares. Arthritis & rheumatology (Hoboken, NJ) 2019; 71: 1580-1586. 2019/03/26. DOI: 10.1002/art.40896. |
| 311 | Zhang Y, Pan R, Xu Y, et al. Treatment of refractory gout with TNF-α antagonist etanercept combined with febuxostat. Ann Palliat Med 2020; 9: 4332-4338. 2020/12/12. DOI: 10.21037/apm-20-2072. |
| 312 | Zhou M, Hua L, Wang YF, et al. Oral Huzhang granules for the treatment of acute gouty arthritis: protocol for a double-blind, randomized, controlled trial. Trials 2022; 23: 248. 2022/04/03. DOI: 10.1186/s13063-022-06188-x. |
| 313 | Late-Breaking Abstracts: American College of Rheumatology 2009 Annual Scientific Meeting. Arthritis and Rheumatism 2009; 60. Conference Review. |
| 314 | Proceedings of the 25th European Paediatric Rheumatology Congress, PReS 2018. Pediatric Rheumatology 2018; 16. Conference Review. |
| 315 | British Society for Rheumatology Annual Conference 2023 Abstracts. Rheumatology (United Kingdom) 2023; 62. Conference Review. |
| 316 | Abdellatif AA and Elkhalili N. Management of gouty arthritis in patients with chronic kidney disease. American journal of therapeutics 2012. Article in Press. DOI: 10.1097/MJT.0b013e318250f83d. |
| 317 | Abdellatif AA and Elkhalili N. Management of gouty arthritis in patients with chronic kidney disease. American journal of therapeutics 2014; 21: 523-534. Review. DOI: 10.1097/MJT.0b013e318250f83d. |
| 318 | Abhishek A. Management of CPPD disease. Annals of the Rheumatic Diseases 2019; 78: 2-3. Conference Abstract. DOI: 10.1136/annrheumdis-2019-eular.8410. |
| 319 | Akgul O, Kilic E, Kilic G, et al. Efficacy and safety of biologic treatments in Familial Mediterranean Fever. American Journal of the Medical Sciences 2013; 346: 137-141. Review. DOI: 10.1097/MAJ.0b013e318277083b. |
| 320 | Alten R, Bardin T, Bloch M, et al. Safety and efficacy of canakinumab in frequently flaring gouty arthritis patients who are contraindicated, intolerant or unresponsive to non-steroidal anti-inflammatory drugs and/or colchicine: Results from 3 years follow-up. Annals of the Rheumatic Diseases 2015; 74: 544. Conference Abstract. DOI: 10.1136/annrheumdis-2015-eular.2199. |
| 321 | Alten R, Bloch M, Bardin T, et al. Efficacy and safety of canakinumab vs triamcinolone acetonide in persistent or elderly gouty arthritis patients. Annals of the Rheumatic Disease 2013; 71. Conference Abstract. DOI: 10.1136/annrheumdis-2012-eular.1080. |
| 322 | Alten R, Schlesinger N, So A, et al. Long-term safety and efficacy of canakinumab vs triamcinolone acetonide in patients with frequent acute gouty arthritis attacks who are contraindicated, intolerant or unresponsive to NSAIDs and/or colchicine. Annals of the Rheumatic Diseases 2014; 73. Conference Abstract. DOI: 10.1136/annrheumdis-2014-eular.1807. |
| 323 | Alvarado JA, Lopez JM, Bardan AC, et al. Efficacy of atorvastatin versus colchicine in decreasing biomarkers of myocardial damage in patients with severe rheumatoid arthritis. Annals of the Rheumatic Diseases 2020; 79: 600-601. Conference Abstract. DOI: 10.1136/annrheumdis-2020-eular.2513. |
| 324 | Amarilyo G, Rothman D, Manthiram K, et al. Consensus treatment plans for periodic fever, aphthous stomatitis, pharyngitis and adenitis syndrome (PFAPA): A framework to evaluate treatment responses from the childhood arthritis and rheumatology research alliance (CARRA) PFAPA work group. Pediatric Rheumatology 2020; 18. Article. DOI: 10.1186/s12969-020-00424-x. |
| 325 | Aviel YB, Rawan S, Fahoum S, et al. Discontinuation of Colchicine Therapy in Children With Familial Mediterranean Fever. Journal of Rheumatology 2021; 48: 1732-1735. Article. DOI: 10.3899/jrheum.201158. |
| 326 | Banerjee M, Pal R, Maisnam I, et al. Serum uric acid lowering and effects of sodium-glucose cotransporter-2 inhibitors on gout: A meta-analysis and meta-regression of randomized controlled trials. Diabetes, Obesity and Metabolism 2023; 25: 2697-2703. Article. DOI: 10.1111/dom.15157. |
| 327 | Bardin T, So A, Alten R, et al. Efficacy and safety of canakinumab vs triamcinolone acetonide in patients with gouty arthritis unable to use nonsteroidal anti-inflammatory drugs and colchicine, and on stable urate lowering therapy (ULT) or unable to use ULT. Arthritis and Rheumatism 2012; 64: S811-S812. Conference Abstract. DOI: 10.1002/art.37735. |
| 328 | Barry A, Helget LN, Androsenko M, et al. Comparison of Gout Flares With the Initiation of Treat-to-Target Allopurinol and Febuxostat: A Post-Hoc Analysis of a Randomized Multicenter Trial. Arthritis and Rheumatology 2024. Article in Press. DOI: 10.1002/art.42927. |
| 329 | Baspinar SN, Alkan A, Yuzbasioglu MB, et al. APPENDICITIS STILL A MISDIAGNOSIS for FMF PATIENTS. Annals of the Rheumatic Diseases 2022; 81: 1767. Conference Abstract. DOI: 10.1136/annrheumdis-2022-eular.5167. |
| 330 | Ben-Chetrit E, Peleg H, Aamar S, et al. The spectrum of MEFV clinical presentations--is it familial Mediterranean fever only? Rheumatology (Oxford, England) 2009; 48: 1455-1459. Review. DOI: 10.1093/rheumatology/kep296. |
| 331 | BenEzra D, Cohen E, Chajek T, et al. Evaluation of conventional therapy versus cyclosporine A in Behcet's syndrome. Transplantation Proceedings 1988; 20: 136-143. Article. |
| 332 | Black S, McKee N, McKnight J, et al. The TICOG Study: Tight Control of Gout-A Randomized, Controlled Trial of Targeted versus Conventional Treatment for Gout Including Ultrasonography. Arthritis and Rheumatology 2022; 74: 3122-3123. Conference Abstract. DOI: 10.1002/art.42355. |
| 333 | Boland EW. NONSPECIFIC ANTI-INFLAMMATORY AGENTS. SOME NOTES ON THEIR PRACTICAL APPLICATION, ESPECIALLY IN RHEUMATIC DISORDERS. California medicine 1964; 100: 145-155. Article. |
| 334 | Bonitsis NG, Altenburg A, Krause L, et al. Current concepts in the treatment of Adamantiades-Behçet's disease. Drugs of the Future 2009; 34: 749-763. Review. DOI: 10.1358/dof.2009.034.09.1416391. |
| 335 | Borstad GC, Bryant LR, Abel MP, et al. Colchicine for prophylaxis of acute flares when initiating allopurinol for chronic gouty arthritis. Journal of Rheumatology 2004; 31: 2429-2432. Article. |
| 336 | Botson J, Peloso PM, Obermeyer K, et al. Pegloticase response improvement by co-treatment with methotrexate: Results from the mirror open-label clinical trial in patients with uncontrolled gout. Annals of the Rheumatic Diseases 2020; 79: 446. Conference Abstract. DOI: 10.1136/annrheumdis-2020-eular.3932. |
| 337 | Brown JP, So A, Dikranian A, et al. Long-term efficacy and safety of canakinumab versus triamcinolone acetonide in acute gouty arthritis patients. Arthritis and Rheumatism 2011; 63. Conference Abstract. |
| 338 | Calguneri M, Kiraz S, Ertenli I, et al. The effect of prophylactic penicillin treatment on the course of arthritis episodes in patients with Behcet's disease: A randomized clinical trial. Arthritis and Rheumatism 1996; 39: 2062-2065. Article. DOI: 10.1002/art.1780391216. |
| 339 | Carter J, Patelli M, Anderson S, et al. An assessment of chronic synovial-based inflammation and its role with serum urate levels. Annals of the Rheumatic Diseases 2013; 72. Conference Abstract. DOI: 10.1136/annrheumdis-2013-eular.2077. |
| 340 | Cheng Y, Huang XP, Tang Y, et al. Effects of evodiamine on ROS/TXNIP/NLRP3 pathway against gouty arthritis. Naunyn-Schmiedeberg's archives of pharmacology 2024; 397: 1015-1023. Article. DOI: 10.1007/s00210-023-02654-8. |
| 341 | Chhibar PS and Ehresmann G. Anakinra as a successful treatment of idiopathic recurrent pericarditis: Taper or not to taper? case series at the university of Southern California. Annals of the Rheumatic Diseases 2017; 76: 421-422. Conference Abstract. DOI: 10.1136/annrheumdis-2017-eular.1141. |
| 342 | Colon-Cortes Y, Hasan MA and Thatayatikom A. Atypical presentation of an uncommon disease: A case of idiopathic chronic eosinophilic pneumonia. American Journal of Respiratory and Critical Care Medicine 2017; 195. Conference Abstract. DOI: 10.1164/ajrccmconference.2017.C66. |
| 343 | Conley B, Bunzli S, Bullen J, et al. What are the core recommendations for gout management in first line and specialist care? Systematic review of clinical practice guidelines. BMC rheumatology 2023; 7. Article. DOI: 10.1186/s41927-023-00335-w. |
| 344 | Cronstein BN and Terkeltaub R. The inflammatory process of gout and its treatment. Arthritis Research and Therapy 2006; 8. Review. DOI: 10.1186/ar1908. |
| 345 | Cronstein BN and Terkeltaub R. The inflammatory process of gout and its treatment. Arthritis Research and Therapy 2006; 8. Review. DOI: 10.1186/ar1908. |
| 346 | Cronstein BN and Terkeltaub R. The inflammatory process of gout and its treatment. Arthritis research & therapy 2006; 8: S3. Review. |
| 347 | Cruz M, Villanueva C, Palileo-Villanueva L, et al. Should prophylaxis with colchicine be given to adult patients with gout when initiating uratelowering therapy: An Evidence Summary. International Journal of Rheumatic Diseases 2021; 24: 10. Conference Abstract. DOI: 10.1111/1756-185X.14199. |
| 348 | Curiel RV and Guzman NJ. Challenges Associated with the Management of Gouty Arthritis in Patients with Chronic Kidney Disease: A Systematic Review. Seminars in arthritis and rheumatism 2012; 42: 166-178. Review. DOI: 10.1016/j.semarthrit.2012.03.013. |
| 349 | Dai H, Lyu S, Wang D, et al. Therapeutic effect of sunflower powder active ingredients on hyperuricemia in mice. Journal of Jilin University Medicine Edition 2018; 44: 327-331. Article. DOI: 10.13481/j.1671-587x.20180222. |
| 350 | Darren A, Levasseur K and Chandratre P. Myositis as an idiosyncratic drug reaction to leflunomide. Rheumatology Advances in Practice 2019; 3: i18. Conference Abstract. DOI: 10.1093/rap/rkz023.002. |
| 351 | Davis C, Ruediger C, Dyer K, et al. Colchicine is not effective for reducing osteoarthritic hand pain compared to placebo: a randomised, placebo-controlled trial (COLAH). Annals of the Rheumatic Diseases 2020; 79: 797-798. Conference Abstract. DOI: 10.1136/annrheumdis-2020-eular.4040. |
| 352 | Demiroglu H, Özcebe OI, Barista I, et al. Interferon alfa-2b, colchicine, and benzathine penicillin versus colchicine and benzathine penicillin in Behcet's disease: A randomised trial (Retraction in: Lancet (2000) 356:9238 (1292)). Lancet 2000; 355: 605-609. Article. DOI: 10.1016/S0140-6736(99)05131-4. |
| 353 | Dundar HA, Turkucar S, Acari C, et al. Familial cold autoinflammatory syndrome-2 (FCAS-2), presenting only with recurrent arthritis. Pediatric Rheumatology 2018; 16. Conference Abstract. DOI: 10.1186/s12969-018-0265-6. |
| 354 | Esty B and Firszt R. Resolution of treatment-resistant recurrent aphthous stomatitis with colchicine in a patient with muckle-wells syndrome: A case report. Journal of Allergy and Clinical Immunology 2015; 135: AB181. Conference Abstract. |
| 355 | Finckh A, Mc Carthy GM, Madigan A, et al. Methotrexate in chronic-recurrent calcium pyrophosphate deposition disease: No significant effect in a randomized crossover trial. Arthritis Research and Therapy 2014; 16. Article. DOI: 10.1186/s13075-014-0458-4. |
| 356 | Garanin AA, Novichkova NL and Lebedev PA. PROSPECTS OF ANTI-INFLAMMATORY AND URATE-LOWERING THERAPY OF GOUT: A VECTOR FROM THE PAST TO THE FUTURE. Nauchno-Prakticheskaya Revmatologiya 2022; 60: 205-213. Article. DOI: 10.47360/1995-4484-2022-205-213. |
| 357 | Genvo MF, Faure M and Thivolet J. Thalidomide and thalidomide with colchicine in the treatment of aphthosis. Recurrent mucocutaneous aphthae and Behcet's disease. Dermatologica 1984; 168: 182-188. Article. |
| 358 | Gertz MA and Kyle RA. Amyloidosis: Prognosis and treatment. Seminars in arthritis and rheumatism 1994; 24: 124-138. Article. DOI: 10.1016/S0049-0172(05)80006-X. |
| 359 | Grygiel-Górniak B, Masiero E, Nevaneeth BC, et al. Rheumatic Diseases in Reproductive Age—the Possibilities and the Risks. Reproductive Sciences 2023; 30: 111-123. Review. DOI: 10.1007/s43032-022-00901-6. |
| 360 | Guillot X, Tordi N, Laheurte C, et al. Local cryotherapy (pulsed CO2 or ice) decreases IL-6, IL-1β and VEGF synovial levels in knee arthritis. Annals of the Rheumatic Diseases 2016; 75: 896. Conference Abstract. DOI: 10.1136/annrheumdis-2016-eular.1699. |
| 361 | Gul A, Ozdogan H, Erer B, et al. Efficacy and safety of canakinumab in adults with colchicine resistant familial mediterranean fever. Arthritis and Rheumatism 2012; 64: S322. Conference Abstract. DOI: 10.1002/art.37735. |
| 362 | Gupta NM, Panginikkod S, Rawal H, et al. Recent advances in the treatment of chronic tophaceous gout. Journal of general internal medicine 2017; 32: S578. Conference Abstract. |
| 363 | Haar NT, Lachmann H, Özen S, et al. Treatment of autoinflammatory diseases: Results from the Eurofever Registry and a literature review. Annals of the Rheumatic Diseases 2013; 72: 678-685. Article. DOI: 10.1136/annrheumdis-2011-201268. |
| 364 | Hashkes P and Huang B. The familial mediterranean fever (FMF) 50 score: Does it work in a controlled clinical trial? Re-analysis of the trial of rilonacept for patients with colchicine resistant or intolerant FMF. Pediatric Rheumatology 2015; 13: 234DUMMY. Conference Abstract. |
| 365 | Hashkes PJ and Huang B. The familial mediterranean fever (FMF) 50 score: Does it work in a controlled clinical trial? re-analysis of the trial of rilonacept for patients with colchicine-resistant or intolerant FMF. Israel Medical Association Journal 2015; 17: 137-140. Article. |
| 366 | He Y, Dai C, Shen J, et al. Effect of Baihu and Guizhi decoction in acute gouty arthritis: study protocol for a randomized controlled trial. Trials 2022; 23. Article. DOI: 10.1186/s13063-022-06194-z. |
| 367 | Hidayat R, Reagan M and Hayati L. Tempuyung leaves (Sonchus arvensis) ameliorates monosodium urate crystal-induced gouty arthritis in rats through anti-inflammatory effects. Open Access Macedonian Journal of Medical Sciences 2020; 8: 220-224. Article. DOI: 10.3889/oamjms.2020.3801. |
| 368 | Higginbottom A, Blackburn S, Campbell L, et al. Celebrating ten years of successful patient involvement in research of inflammatory conditions. Annals of the Rheumatic Diseases 2017; 76: 1552. Conference Abstract. DOI: 10.1136/annrheumdis-2017-eular.3144. |
| 369 | Hill EM, Sky K, Sit M, et al. Does starting allopurinol prolong acute treated gout? a randomized clinical trial. Journal of Clinical Rheumatology 2015; 21: 120-125. Article. DOI: 10.1097/RHU.0000000000000235. |
| 370 | Horsley A, Helm JM, Brennan A, et al. Is gout the next emerging complication for adults with CF? Pediatric Pulmonology 2010; 45: 417. Conference Abstract. DOI: 10.1002/(ISSN)1099-0496. |
| 371 | Jansen T, Kluck V, Janssen M, et al. The first phase 2a proof-of-concept study of a selective NLRP3 inflammasome inhibitor, dapansutrile™ (OLT1177™), in acute gout. Arthritis and Rheumatology 2019; 71: 5034-5036. Conference Abstract. DOI: 10.1002/art.41108. |
| 372 | Jansen TL, Klück V, Janssen M, et al. The first phase 2A proof-of-concept study of a selective NLRP3 inflammasome inhibitor, dapansutrile™ (OLT1177™), in acute gout. Annals of the Rheumatic Diseases 2019; 78: A70-A71. Conference Abstract. DOI: 10.1136/annrheumdis-2018-EWRR2019.142. |
| 373 | Janssens HJ, Janssen M, van de Lisdonk EH, et al. Use of oral prednisolone or naproxen for the treatment of gout arthritis: a double-blind, randomised equivalence trial. The Lancet 2008; 371: 1854-1860. Article. DOI: 10.1016/S0140-6736(08)60799-0. |
| 374 | Jones A, Paterson-Brown L and Shivamurthy V. A DIAGNOSIS OF BEHCET'S DISEASE WITH SECONDARY ANTIPHOSPHOLIPID SYNDROME. Rheumatology Advances in Practice 2021; 5: i10-i11. Conference Abstract. DOI: 10.1093/rap/rkab067.015. |
| 375 | Jones G, Crotty M and Brooks P. Psoriatic arthritis: A quantitative overview of therapeutic options. British journal of rheumatology 1997; 36: 95-99. Article. |
| 376 | Jones G, Crotty M and Brooks P. Interventions for psoriatic arthritis. Cochrane database of systematic reviews (Online) 2000: CD000212. Review. |
| 377 | Jones G, Crotty M and Brooks P. Interventions for psoriatic arthritis. Cochrane database of systematic reviews (Online) 2000: CD000212. Review. |
| 378 | Karimzadeh H, Nazari J, Mottaghi P, et al. Different duration of colchicine for preventing recurrence of gouty arthritis. Journal of Research in Medical Sciences 2006; 11: 104-107. Article. |
| 379 | Kemta Lekpa F and Kwimatouo Lekpa AF. Evidence of the use of colchicine are more numerous in cardiology and hepato-gastroenterology than in rheumatology: A systematic literature review of clinical trials. Annals of the Rheumatic Diseases 2016; 75: 375-376. Conference Abstract. DOI: 10.1136/annrheumdis-2016-eular.2588. |
| 380 | Khanna I and Bhalla A. When the fever broke-a curious case of periodic fever syndrome. Journal of general internal medicine 2020; 35: S619. Conference Abstract. DOI: 10.1007/s11606-020-05890-3. |
| 381 | Khanna P and Beal R. The Impact of Topically Applied pH Modulator on Acute Inflammatory Pain, Serum Calcium, and C-Reactive Protein (CRP) during an Acute Gout Flare-a Phase 2a Randomized, Double-Blind, Placebo-Controlled Study. Arthritis and Rheumatology 2022; 74: 3529-3531. Conference Abstract. DOI: 10.1002/art.42355. |
| 382 | Khanna P and Beal R. PHASE 2A, RANDOMIZED, DOUBLE-BLIND, PLACEBO-CONTROLLED STUDY of the EFFICACY and SAFETY of A TRANSDERMAL ALKALINIZING and PAIN-RELIEVING TREATMENT for REDUCING PAIN ASSOCIATED with AN ACUTE GOUT FLARE. Annals of the Rheumatic Diseases 2022; 81: 112. Conference Abstract. DOI: 10.1136/annrheumdis-2022-eular.4909. |
| 383 | Khanna P, Singh MK, FitzGerald JD, et al. Pharmacological treatment of acute gout: A systematic review. Arthritis and Rheumatism 2011; 63. Conference Abstract. |
| 384 | Khanna PP, Gladue HS, Singh MK, et al. Treatment of acute gout: A systematic review. Seminars in arthritis and rheumatism 2014; 44: 31-38. Review. DOI: 10.1016/j.semarthrit.2014.02.003. |
| 385 | Kötter I, Deuter C, Henes J, et al. Canakinumab for Behçet's disease resistant to standard treatment (CanBeDisT)-an open-label single center pilot study. Clinical and Experimental Rheumatology 2018; 36: S187. Conference Abstract. |
| 386 | Kulaksiz B, Egeli BH, Aydin O, et al. Is exon 2 associated with FMF or a new disease. Arthritis and Rheumatology 2019; 71: 2162-2164. Conference Abstract. DOI: 10.1002/art.41108. |
| 387 | Kuppuraju R, May P, Millliken T, et al. TREATMENT OF GOUT AND ITS EFFECTS ON METABOLIC SYNDROME. Annals of the Rheumatic Diseases 2023; 82: 521-522. Conference Abstract. DOI: 10.1136/annrheumdis-2023-eular.3206. |
| 388 | Laosuksri P, Phrintrakul N, Gumtorntip W, et al. Non-loading versus loading low-dose colchicine in acute crystal-associated arthritis: A double-blinded randomized controlled study. International Journal of Rheumatic Diseases 2023; 26: 2478-2488. Article. DOI: 10.1111/1756-185X.14943. |
| 389 | Leccese P, Ozguler Y, Christensen R, et al. A systematic literature review on the treatment of skin, mucosa and joint involvement of Behçet's syndrome informing the Eular recommendations for the management of Behçet's syndrome. Arthritis and Rheumatology 2016; 68: 3971-3972. Conference Abstract. DOI: 10.1002/art.39977. |
| 390 | Leccese P, Ozguler Y, Christensen R, et al. A systematic literature review on the treatment of skin, mucosa and joint involvement of behçet's syndrome informing the eular recommendations for the management of behçet's syndrome. Annals of the Rheumatic Diseases 2016; 75: 798. Conference Abstract. DOI: 10.1136/annrheumdis-2016-eular.5609. |
| 391 | Leccese P, Ozguler Y, Christensen R, et al. Systematic review on the treatment of skin, mucosa and joint involvement of Behçet's syndrome informing the eular recommendations for the management of Behçet's syndrome. Rheumatology (United Kingdom) 2017; 56: iii89. Conference Abstract. DOI: 10.1093/rheumatology/kex098. |
| 392 | Lehmann A, Schneck L, Keenan RT, et al. Colchicine use is associated with decreased diagnosis of myocardial infarction (MI) and trend toward reduction of all-cause mortality and c-reactive protein (CRP) levels: Insights from the NYVA gout cohort. Arthritis and Rheumatism 2010; 62: 148. Conference Abstract. DOI: 10.1002/art.27917. |
| 393 | Leung YY, Huebner JL, Wong B, et al. Association of synovial fluid inflammatory biomarkers and knee pain. Osteoarthritis and Cartilage 2016; 24: S73. Conference Abstract. |
| 394 | Leventhal LJ, Levin RW and Bomalaski JS. Peripheral arthrocentesis in the work-up of acute low back pain. Archives of physical medicine and rehabilitation 1990; 71: 253-254. Article. |
| 395 | Liang H, Wu Y, Zhang W, et al. Efficacy and Safety of Acupuncture Combined with Herbal Medicine in Treating Gouty Arthritis: Meta-Analysis of Randomized Controlled Trials. Evidence-based Complementary and Alternative Medicine 2021; 2021. Article. DOI: 10.1155/2021/8161731. |
| 396 | Lin FF, Liu SM, Zhou Q, et al. Effects of total saponins from rhizoma dioscorea nipponica on biomarkers in serum of gouty arthritis rats. Chinese Journal of New Drugs 2017; 26: 2840-2845. Article. |
| 397 | Lin N, Dai Q, Zhang Y, et al. Chinese classical decoction Wuwei Xiaodu Drink alleviates gout arthritis by suppressing NLRP3-Mediated inflammation. Frontiers in Pharmacology 2024; 15. Article. DOI: 10.3389/fphar.2024.1388753. |
| 398 | Liu P, Chen Y, Wang B, et al. Expression of microRNAs in the plasma of patients with acute gouty arthritis and the effects of colchicine and etoricoxib on the differential expression of microRNAs. Archives of Medical Science 2019; 15: 1047-1055. Article. DOI: 10.5114/aoms.2018.75502. |
| 399 | Liu R, Hu XC and Bian XH. Effect of Qingxi Softshell Turtle Peptides on Gout Arthritis in Rats Caused by Sodium Urate. Chinese Journal of Pharmaceutical Biotechnology 2021; 28: 579-584. Article. DOI: 10.19526/j.cnki.1005-8915.20210605. |
| 400 | Liu W, Yang HJ, Wu YH, et al. Qinpi decoction intervenes gouty arthritis by regulating the MyD88-dependent TLR signal pathway. International Journal of Rheumatic Diseases 2016; 19: 22. Conference Abstract. DOI: 10.1111/1756-185X.12962. |
| 401 | Liu Y, Li ZC, Chen JB, et al. Therapeutic efficacy of small doses of colchicine combined with glucocorticoid for acute gouty arthritis. Medical Journal of Chinese People's Liberation Army 2015; 40: 652-655. Article. DOI: 10.11855/j.issn.0577-7402.2015.08.10. |
| 402 | Lu H, Liu W, Fan Y, et al. Efficacy and Safety of Qinpi Tongfeng Formula Combined with Bloodletting Therapy in the Treatment of Acute Gouty Arthritis: A Study Protocol for a Randomized Controlled Trial. Evidence-based Complementary and Alternative Medicine 2022; 2022. Article. DOI: 10.1155/2022/3147319. |
| 403 | Lu R, Cao Y, Lei X, et al. Danggui Sinitang Mitigates Gouty Arthritis in Rats by Regulating Autophagy via PI3K/Akt/mTOR Signaling Pathway. Chinese Journal of Experimental Traditional Medical Formulae 2023; 29: 78-84. Article. DOI: 10.13422/j.cnki.syfjx.20230244. |
| 404 | Maffi M, De Mattia G, Mazzoni MR, et al. Calcification of Joints and Arteries (CALJA) Is a Rare Cause of Arthritis and Lower Limb Ischemia: Case Report and Literature Review. SN Comprehensive Clinical Medicine 2023; 5. Article. DOI: 10.1007/s42399-023-01485-1. |
| 405 | Makay B and Ünsal E. P02-008-Dramatic response to canakinumab in MKD. Pediatric Rheumatology 2013; 11. Conference Abstract. DOI: 10.1186/1546-0096-11-S1-A115. |
| 406 | Maly M, Viktorová J, Ruml T, et al. Anti-inflammatory potential of cannabinoid extracts. Cardiovascular Research 2022; 118: ii52. Conference Abstract. |
| 407 | McKendry RJR, Kraag G, Seigel S, et al. Therapeutic value of colchicine in the treatment of patients with psoriatic arthritis. Annals of the Rheumatic Diseases 1993; 52: 826-828. Article. DOI: 10.1136/ard.52.11.826. |
| 408 | McLaughlin D, McKenna D, Campbell C, et al. A CASE OF PYOGENIC ARTHRITIS, PYODERMA GANGRENOSUM AND ACNE SYNDROME. Rheumatology Advances in Practice 2021; 5: i12-i13. Conference Abstract. DOI: 10.1093/rap/rkab067.018. |
| 409 | Messina MR, Costanzo GAML, Barca MP, et al. Anakinra as a diagnostic and therapeutic trial for Undifferentiated Autoinflammatory Syndromes. Management of three clinical cases. Allergy: European Journal of Allergy and Clinical Immunology 2020; 75: 320-321. Conference Abstract. DOI: 10.1111/all.14508. |
| 410 | Nidorf SM and Thompson PL. Why Colchicine Should Be Considered for Secondary Prevention of Atherosclerosis: An Overview. Clinical therapeutics 2019; 41: 41-48. Review. DOI: 10.1016/j.clinthera.2018.11.016. |
| 411 | Nilsson F, Norberg B, Frederiksen B, et al. The role of the MA-sensitive leukocyte chemotaxis in rheumatoid arthritis. A randomized double-blind clinical trial of griseofulvin treatment. Scandinavian Journal of Rheumatology 1983; 12: 113-118. Article. |
| 412 | Oliviero F, Galozzi P, Scanu A, et al. Polydatin prevents calcium pyrophosphate crystal-induced arthritis in mice. Nutrients 2021; 13: 1-13. Article. DOI: 10.3390/nu13030929. |
| 413 | Oliviero F, Galuppini F, Scanu A, et al. Polydatin prevents calcium pyrophosphate crystal-induced arthritis in mice. Annals of the Rheumatic Diseases 2020; 79: 108-109. Conference Abstract. DOI: 10.1136/annrheumdis-2020-eular.2124. |
| 414 | Otani K, Watanabe T, Nadatani Y, et al. EFFECT OF COLCHICINE TREATMENT ON SEVERE NSAID-INDUCED SMALL INTESTINAL DAMAGE: A PILOT STUDY. Gastrointestinal Endoscopy 2020; 91: AB613-AB614. Conference Abstract. DOI: 10.1016/j.gie.2020.03.3720. |
| 415 | Ozen S. New interest in an old disease: Familial Mediterranean fever. Clinical and Experimental Rheumatology 1999; 17: 745-749. Review. |
| 416 | Ozen S. Familial mediterranean fever: Revisiting an ancient disease. European journal of pediatrics 2003; 162: 449-454. Review. DOI: 10.1007/s00431-003-1223-x. |
| 417 | Parisa N, Hidayat R, Maritska Z, et al. Evaluation of the anti-gout effect of Sonchus Arvensis on monosodium urate crystal-induced gout arthritis via anti-inflammatory action - an in vivo study. Medicine and pharmacy reports 2021; 94: 358-365. Article. DOI: 10.15386/mpr-1959. |
| 418 | Parperis K, Papachristodoulou E, Kakoullis L, et al. Management of calcium pyrophosphate crystal deposition disease: A systematic review. Seminars in arthritis and rheumatism 2021; 51: 84-94. Review. DOI: 10.1016/j.semarthrit.2020.10.005. |
| 419 | Pascart T, Robinet P, Ottaviani S, et al. Evaluating the safety and short-term equivalence of colchicine versus prednisone in older patients with acute calcium pyrophosphate crystal arthritis (COLCHICORT): an open-label, multicentre, randomised trial. The Lancet Rheumatology 2023; 5: e523-e531. Article. DOI: 10.1016/S2665-9913(23)00165-0. |
| 420 | Pascart T, Robinet P, Ottaviani S, et al. Colchicine or Prednisone for the Treatment of Acute Calcium Pyrophosphate Deposition Arthritis: A Multicenter Randomized Controlled Trial. Arthritis and Rheumatology 2022; 74: 3131-3134. Conference Abstract. DOI: 10.1002/art.42355. |
| 421 | Pascual E, Andrés M and Sivera F. Methotrexate: Should it still be considered for chronic calcium pyrophosphate crystal disease? Arthritis Research and Therapy 2015; 17. Editorial. DOI: 10.1186/s13075-015-0598-1. |
| 422 | Pu MJ, Yao CJ, Liu LM, et al. Traditional Chinese medicine for gouty arthritis: A protocol for meta-analysis. Medicine (United States) 2021; 100: E23699. Review. DOI: 10.1097/MD.0000000000023699. |
| 423 | Qian X, Jiang Y, Luo Y, et al. The Anti-hyperuricemia and Anti-inflammatory Effects of Atractylodes Macrocephala in Hyperuricemia and Gouty Arthritis Rat Models. Combinatorial Chemistry and High Throughput Screening 2023; 26: 950-964. Article. DOI: 10.2174/1386207325666220603101540. |
| 424 | Qiao TX, Tang CL, Qiu L, et al. Cathepsin-B involved in effect of electroacupuncture by inhibiting the activation of NLRP3 inflammasome in rats with acute gouty arthritis. Zhen ci yan jiu = Acupuncture research 2021; 46: 295-300. Article. DOI: 10.13702/j.1000-0607.200240. |
| 425 | Qiu L, Tang CL, Huang SQ, et al. Effect of Electroacupuncture on Synovial M 1/M 2 Macrophage Polarization in Rats with Acute Gouty Arthritis. Zhen ci yan jiu = Acupuncture research 2018; 43: 767-772. Article. DOI: 10.13702/j.1000-0607.180498. |
| 426 | Ramesh R, Sriram S, Kumudha M, et al. Interleukin 1B levels in acute gout-before and after colchicine-a randomised prospective interventional study. International Journal of Rheumatic Diseases 2015; 18: 17-18. Conference Abstract. |
| 427 | Ramos-Bello D, Alvarez-Quiroga C, Barragán-Pickens GA, et al. Effect of methotrexate (MTX), and MTX plus colchicine (CCH) on the expression and activity of NLRP3 inflammasome in patients with early rheumatoid arthritis (RA). Arthritis and Rheumatology 2017; 69. Conference Abstract. |
| 428 | Rodnan GP, Robin JA and Tolchin S. Efficacy of a single daily dose of allopurinol in gouty hyperuricemia. Israel Journal of Medical Sciences 1973; 9: 1111-1112. |
| 429 | Rongmin D, Ningyi L, Hongshu X, et al. Regulating serum inflammatory factors and synovial cell function of gouty arthritis model rats by alcohol extract of gardneria angustifolia. Chinese Journal of Tissue Engineering Research 2021; 25: 2681-2686. Article. DOI: 10.3969/j.issn.2095-4344.3193. |
| 430 | Saag K, So A, Khanna P, et al. A randomized, phase 2 study evaluating the efficacy and safety of Anakinra in difficult-to-treat acute gouty arthritis: The Anago study. Arthritis and Rheumatology 2019; 71: 2136-2138. Conference Abstract. DOI: 10.1002/art.41108. |
| 431 | Saag K, So A, Khanna P, et al. A randomized, phase 2 study evaluating the efficacy and safety of anakinra in difficultto-treat acute gouty arthritis: The Anago study. Annals of the Rheumatic Diseases 2020; 79: 442. Conference Abstract. DOI: 10.1136/annrheumdis-2020-eular.3766. |
| 432 | Saenz A, Ausejo M, Shea B, et al. Pharmacotherapy for Behcet's syndrome. Cochrane database of systematic reviews (Online) 2000: CD001084. Review. |
| 433 | Salih MS, Mlatoum HA, Leung E, et al. Colchicine for prevention of pericarditis recurrence: Metaanalysis of randomized controlled trials. Cardiology (Switzerland) 2015; 131: 118. Conference Abstract. |
| 434 | Sandhu T, Tieng A, Chilimuri S, et al. A case control study to evaluate the impact of colchicine on patients admitted to the hospital with moderate to severe covid-19 infection. Canadian Journal of Infectious Diseases and Medical Microbiology 2020; 2020. Article. DOI: 10.1155/2020/8865954. |
| 435 | Satpanich P and Manavathongchai S. Early versus late allopurinol initiation in an acute gout attack: A randomized controlled trial (ELAG). International Journal of Rheumatic Diseases 2020; 23: 178-179. Conference Abstract. DOI: 10.1111/1756-185X.13985. |
| 436 | Schlesinger N. Overview of the management of acute gout and the role of adrenocorticotropic hormone. Drugs 2008; 68: 407-415. Review. DOI: 10.2165/00003495-200868040-00002. |
| 437 | Schlesinger N, Alten R, Bardin T, et al. Effect of canakinumab vs triamcinolone acetonide for treatment of gouty arthritis in patients who are unable to use NSAIDs and colchicine or with severe gouty arthritis. Annals of the Rheumatic Disease 2013; 71. Conference Abstract. DOI: 10.1136/annrheumdis-2012-eular.2826. |
| 438 | Schlesinger N, Alten R, Bardin T, et al. Canakinumab in frequently flaring gouty arthritis patients, contraindicated, intolerant or unresponsive to non-steriodal anti-inflammatory drugs and/or colchicine: Safety and efficacy results from long term follow-up. Arthritis and Rheumatology 2014; 66: S66-S67. Conference Abstract. DOI: 10.1002/art.38914. |
| 439 | Schlesinger N, Brown JP, Bardin T, et al. Comparison of pain intensity, incidence of new flares, safety and tolerability of canakinumab vs triamcinolone acetonide in gouty arthritis patients with cardiovascular diseases or with cardiovascular risk factors. Arthritis and Rheumatism 2011; 63. Conference Abstract. |
| 440 | Schlesinger N, De Meulemeester M, Pikhlak A, et al. Canakinumab relieves symptoms of acute flares and improves health-related quality of life in patients with difficult-to-treat Gouty Arthritis by suppressing inflammation: Results of a randomized, dose-ranging study. Arthritis Research and Therapy 2010; 13. Article. DOI: 10.1186/ar3297. |
| 441 | Schlesinger N, De Meulemeester M, Shamim T, et al. Canakinumab vs triamcinolone acetonide for treatment of acute flares and prevention of recurrent flares in “difficult to treat” gouty arthritis. Journal of Clinical Rheumatology 2010; 16: S23. Conference Abstract. DOI: 10.1097/RHU.0b013e3181db7b64. |
| 442 | Schlesinger N, DeMeulemeester M, Shamim T, et al. Canakinumab Vs. Triamcinolone acetonide for treatment of 'difficult to treat' gouty arthritis patients. Internal Medicine Journal 2010; 40: 49. Conference Abstract. DOI: 10.1111/j.1445-5994.2010.02186.x. |
| 443 | Schlesinger N, Detry MA, Holland BK, et al. Local ice therapy during bouts of acute gouty arthritis. Journal of Rheumatology 2002; 29: 331-334. Article. |
| 444 | Schlesinger N, Lin H, De Meulemeester M, et al. Efficacy of canakinumab (ACZ885), a fully human anti-interleukin-1beta monoclonal antibody, in the prevention of flares in gout patients initiating allopurinol therapy. Rheumatology 2011; 50: iii85-iii86. Conference Abstract. |
| 445 | Schlesinger N, Lin HY, De Meulemeester M, et al. Efficacy of canakinumab (ACZ885), a fully human anti-interleukin (IL)-1beta monoclonal antibody, in the prevention of flares in Gout patients initiating allopurinol therapy. Arthritis and Rheumatism 2010; 62: 2087. Conference Abstract. DOI: 10.1002/art.29852. |
| 446 | Schlesinger N, Rudolph L, Lin Taipei H, et al. Efficacy of canakinumab (acz885), a fully human anti-interleukin (IL)-1beta monoclonal antibody, in the prevention of flares in gout patients initiating allopurinol therapy. Journal of Investigative Medicine 2011; 59: 710-711. Conference Abstract. DOI: 10.231/JIM.0b013e3182152a03. |
| 447 | Schlesinger N, Schumacher R, Catton M, et al. Colchicine for acute gout. Cochrane Database of Systematic Reviews 2006. Review. DOI: 10.1002/14651858.CD006190. |
| 448 | Scopes JW and Hulse JA. Mucocutaneous lymph node syndrome. British medical journal 1977; 1: 511. Letter. |
| 449 | Sharma A, Provenzale D, McKusick A, et al. Interstitial pneumonitis after low-dose methotrexate therapy in primary biliary cirrhosis. Gastroenterology 1994; 107: 266-270. Article. |
| 450 | Shekelle PG, Newberry SJ, FitzGerald JD, et al. Management of gout: A systematic review in support of an American college of physicians clinical practice guideline. Annals of internal medicine 2017; 166: 37-51. Review. DOI: 10.7326/M16-0461. |
| 451 | So A, Alten R, Schumacher HR, et al. Inflammation suppression over 24 weeks in patients with gouty arthritis: Results from two phase-III core and extension studies comparing canakinumab with triamcinolone acetonide. Arthritis and Rheumatism 2011; 63. Conference Abstract. |
| 452 | So A, De Meulemeester M, Pikhlak A, et al. Canakinumab (ACZ885) relieves pain and controls inflammation rapidly in patients with difficult-to-treat gouty arthritis: Comparison with triamcinolone acetonide. Arthritis and Rheumatism 2010; 62: 145. Conference Abstract. DOI: 10.1002/art.27914. |
| 453 | So A, De Meulemeester M, Pikhlak A, et al. Efficacy of canakinumab (ACZ885) compared to triamcinolone acetonide for treatment of acute flares and prevention of recurrent flares in gouty arthritis patients. International Journal of Rheumatic Diseases 2010; 13: 173. Conference Abstract. DOI: 10.1111/j.1756-185X.2010.01508.x. |
| 454 | So A, De Meulemeester M, Pikhlak A, et al. Rapid improvement in health-related quality of life (HRQoL) in gouty arthritis patients treated with canakinumab (ACZ885) compared to triamcinolone acetonide. Arthritis and Rheumatism 2010; 62: 1423. Conference Abstract. DOI: 10.1002/art.29189. |
| 455 | So A, De Meulemeester M, Pikhlak A, et al. Canakinumab (ACZ885) relieves pain and controls inflammation rapidly in patients with difficult-to-treat gouty arthritis: Comparison with triamcinolone acetonide. Rheumatology 2011; 50: iii32. Conference Abstract. |
| 456 | So A, De Meulemeester M, Pikhlak A, et al. Rapid improvement in health-related quality of life in gouty arthritis patients treated with canakinumab (ACZ885) compared to triamcinolone acetonide. Rheumatology 2011; 50: iii85. Conference Abstract. |
| 457 | So A, De Meulemeester M, Pikhlak A, et al. Canakinumab for the treatment of acute flares in difficult-to-treat gouty arthritis: Results of a multicenter, phase II, dose-ranging study. Arthritis and Rheumatism 2010; 62: 3064-3076. Article. DOI: 10.1002/art.27600. |
| 458 | So A, De Meulemeester M, Shamim T, et al. Canakinumab (ACZ885) vs. triamcinolone acetonide for treatment of acute flares and prevention of recurrent flares in gouty arthritis patients refractory to or contraindicated to NSAIDs and/or colchicine. Arthritis and Rheumatism 2009; 60: 3860. Conference Abstract. DOI: 10.1002/art.27256. |
| 459 | So A, DeMeulenmeester M, Yücel E, et al. Canakinumab relieves symptoms of acute flares and improves health-related quality of life (HRQoL) in difficult-to-treat gouty arthritis patients by suppressing inflammation: Results of a randomized, dose-ranging study. Journal of Allergy and Clinical Immunology 2011; 127: AB227. Conference Abstract. DOI: 10.1016/j.jaci.2010.12.903. |
| 460 | So A, Meulemeester MD, Bodalia B, et al. Canakinumab (ACZ885) vs triamcinolone acetonide for treatment of acute flares and prevention of recurrent flares in gouty arthritis patients refractory to or contraindicated to nsaids and/or colchicine. Rheumatology 2010; 49: i60-i61. Conference Abstract. DOI: 10.1093/rheumatology/keq717. |
| 461 | Suchartlikitwong S, Lapumnuaypol K and Getzug T. A rare case of bladder amyloidosis complicating familial mediterranean fever. American Journal of Gastroenterology 2015; 110: S422-S423. Conference Abstract. |
| 462 | Suchartlikitwong S, Lapumnuaypol K and Getzug T. A rare case of bladder amyloidosis complicating familial mediterranean fever. American Journal of Gastroenterology 2015; 110: S422-S423. Conference Abstract. DOI: 10.1038/ajg.2015.270. |
| 463 | Sunkureddi P, Schlesinger N, Kiechle T, et al. Effect of serum urate level on prevention of flare in acute gouty arthritis patients with canakinumab. Annals of the Rheumatic Disease 2013; 71. Conference Abstract. DOI: 10.1136/annrheumdis-2012-eular.1079. |
| 464 | Sunkureddi P, Toth E, Brown J, et al. Canakinumab pre-filled syringe vs triamcinolone acetonide in the treatment of acute gouty arthritis attacks: Results from a post-hoc analysis in difficult-to-treat patients. Annals of the Rheumatic Diseases 2014; 73. Conference Abstract. DOI: 10.1136/annrheumdis-2014-eular.1846. |
| 465 | Sunkureddi P, Toth E, Brown JP, et al. Efficacy and safety of canakinumab pre-filled syringe versus triamcinolone acetonide in acute gouty arthritis patients. Arthritis and Rheumatism 2013; 65: S498. Conference Abstract. DOI: 10.1002/art.38216. |
| 466 | Sunkureddi P, Tóth E, P Brown J, et al. Efficacy and safety of canakinumab in acute gouty arthritis patients with chronic kidney disease stage greater than or equal to 3: A Post-Hoc analysis of 12-week data. Arthritis and Rheumatology 2014; 66: S73-S74. Conference Abstract. DOI: 10.1002/art.38914. |
| 467 | Tetik O, Doral MN, Atay AO, et al. Influence of irrigation solutions combined with colchicine and diclofenac sodium on articular cartilage in a rat model. Knee surgery, sports traumatology, arthroscopy : official journal of the ESSKA 2004; 12: 503-509. Article. |
| 468 | van Durme CMPG, Wechalekar MD, Landewé RBM, et al. Non-steroidal anti-inflammatory drugs for acute gout. Cochrane Database of Systematic Reviews 2021; 2021. Review. DOI: 10.1002/14651858.CD010120.pub3. |
| 469 | Verne GN, Eaker EY, Davis RH, et al. Colchicine is an effective treatment for patients with chronic constipation: An open-label trial. Digestive diseases and sciences 1997; 42: 1959-1963. Article. DOI: 10.1023/A:1018883731556. |
| 470 | Vogel Y, Büchner NJ, Haverkamp T, et al. Familial Mediterranean fever- Rare manifestation without fever or family history. Deutsche Medizinische Wochenschrift 2008; 133: 1621-1624. Article. DOI: 10.1055/s-0028-1082777. |
| 471 | Voulgari PV, Venetsanopoulou AI and Drosos AA. Recent advances in the therapeutic management of calcium pyrophosphate deposition disease. Frontiers in medicine 2024; 11. Review. DOI: 10.3389/fmed.2024.1327715. |
| 472 | Waller A and Jordan KM. Use of febuxostat in the management of gout in the United Kingdom. Therapeutic advances in musculoskeletal disease 2017; 9: 55-64. Review. DOI: 10.1177/1759720X16682010. |
| 473 | Wang GH, Zuo T, Li R, et al. Effect of rebamipide on the acute gouty arthritis in rats induced by monosodium urate crystals. Beijing da xue xue bao Yi xue ban = Journal of Peking University Health sciences 2021; 53: 716-720. Article. |
| 474 | Wang H, Chen ST, Ding XJ, et al. Efficacy and safety of Huzhang Granule, a compound Chinese herbal medicine, for acute gouty arthritis: A double-blind, randomized controlled trial. Journal of Integrative Medicine 2024; 22: 270-278. Article. DOI: 10.1016/j.joim.2024.03.008. |
| 475 | Wang J, Zhong M, Yang K, et al. Intervention Effect and Mechanism of Qingmei Compound on Acute Gouty Arthritis Based on NLRP3 Pathway. Chinese Journal of Experimental Traditional Medical Formulae 2022; 28: 70-76. Article. DOI: 10.13422/j.cnki.syfjx.20221842. |
| 476 | Wang L, Na S and Chen GL. Effect of total saponin of Dioscorea on NALP3 inflammasome signaling pathway with acute gouty in rats. Chinese Pharmacological Bulletin 2017; 33: 354-360. Article. DOI: 10.3969/j.issn.1001-1978.2017.03.013. |
| 477 | Wang P, Ren D, Chen Y, et al. Effect of sodium alginate addition to resveratrol on acute gouty arthritis. Cellular Physiology and Biochemistry 2015; 36: 201-207. Article. DOI: 10.1159/000374064. |
| 478 | Wang YG, Wang L, Li EZ, et al. Chuanhu anti-gout mixture versus colchicine for acute gouty arthritis: A randomized, Double-Blind, Double-Dummy, Non-Inferiority Trial. International Journal of Medical Sciences 2014; 11: 880-885. Article. DOI: 10.7150/ijms.9165. |
| 479 | Wang YG, Wu YQ, Miao ZM, et al. Experimental observation of gout mixture in treatment of gouty arthritis. Chinese Journal of Clinical Rehabilitation 2006; 10: 70-73. Article. |
| 480 | Wei BW, Gao JY, Liu W, et al. Mechanism of Jinteng Qingbi Granules in treatment of acute gouty arthritis. Chinese Traditional and Herbal Drugs 2023; 54: 7086-7095. Article. DOI: 10.7501/j.issn.0253-2670.2023.21.017. |
| 481 | Wu YQ, Hu JH, Zhao ZY, et al. Clinical observation on senile patients with acute gouty arthritis treated by acupoint application. Journal of the American Geriatrics Society 2014; 62: S381. Conference Abstract. DOI: 10.1111/jgs.13075. |
| 482 | Yang DH, Chen HC and Wei JCC. Early lowering of serum uric acid levels in the treatment of gouty arthritis with an acute attack. International Journal of Rheumatic Diseases 2018; 21: 218. Conference Abstract. DOI: 10.1111/1756-185X.13361. |
| 483 | Yang DH, Chen HC and Wei JCC. Early urate-lowering therapy in gouty arthritis with acute flares: a double-blind placebo controlled clinical trial. European Journal of Medical Research 2023; 28. Article. DOI: 10.1186/s40001-022-00982-8. |
| 484 | Yao Q, Su LC, Zhou L, et al. Characterization of a new category of autoinflammatory disease associated with nucleotide oligomerization domain2 gene mutations: An expanded cohort study. Arthritis and Rheumatism 2011; 63. Conference Abstract. |
| 485 | Yu CL, Lu F, Yu DH, et al. Mechanism of acteoside in prevention and treatment of gouty arthritis based on liver metabolomics. Zhongguo Zhongyao Zazhi 2024; 49: 224-231. Article. DOI: 10.19540/j.cnki.cjcmm.20230808.402. |
| 486 | Yu J, Li L, Liu J, et al. Influence of intervention treatment by "heat-clearing and diuresis-promoting" prescription on NALP3, an inflammatory factor in acute gouty arthritis. Journal of orthopaedic surgery and research 2022; 17: 162. Article. DOI: 10.1186/s13018-022-03046-z. |
| 487 | Yurdakul S, Mat C, Tuzun Y, et al. A double-blind trial of colchicine in Behçet's syndrome. Arthritis and Rheumatism 2001; 44: 2686-2692. Article. DOI: 10.1002/1529-0131(200111)44:11<2686::AID-ART448>3.0.CO;2-H. |
| 488 | Zeng WC, Li QH, Tang AJ, et al. EFFICACY and SAFETY of LOW-DOSE RASBURICASE in COMBINATION with CONVENTIONAL URATE-LOWERING THERAPY for REFRACTORY CHRONIC GOUTY ARTHRITIS: A PILOT STUDY in. Annals of the Rheumatic Diseases 2022; 81: 1650. Conference Abstract. DOI: 10.1136/annrheumdis-2022-eular.3450. |
| 489 | Zhang C, Huang X, Luo Y, et al. The effects of electro-acupuncture on the signaling pathway of TLR/MYD88 in ankle joint synovial tissue of acute gouty arthritis rats. Sichuan da xue xue bao Yi xue ban = Journal of Sichuan University Medical science edition 2014; 45: 924-927. Article. |
| 490 | Zhang CN, Huang XK, Luo Y, et al. Effects of electro-acupuncture on expression of triggering receptor expressed on myeloid cells 1 in ankle joint synovial tissue of acute gouty arthritis rats. Nan fang yi ke da xue xue bao = Journal of Southern Medical University 2015; 35: 133-136. Article. |
| 491 | Zhang H, Meng F, Ma W, et al. Protective effect of Tutengcao Granules on acute gouty kidney injury in rats with acute gouty arthritis. Advances in Integrative Medicine 2019; 6: S52-S53. Conference Abstract. DOI: 10.1016/j.aimed.2019.03.154. |
| 492 | Zhang J, Gou B, Wang T, et al. Ultrasound stimulated perfluorobutane microbubbles cavitation enhanced the therapeutic effect of colchicine in rats with acute gouty arthritis. Chinese Journal of Ultrasonography 2024; 33: 609-616. Article. DOI: 10.3760/cma.j.cn131148-20240104-00009. |
| 493 | Zhang J, Sun W, Gao F, et al. Changes of serum uric acid level during acute gout flare and related factors. Frontiers in endocrinology 2023; 14. Article. DOI: 10.3389/fendo.2023.1077059. |
| 494 | Zhang M, Zhang Y, Terkeltaub R, et al. Effect of Dietary and Supplemental Omega-3 Polyunsaturated Fatty Acids on Risk of Recurrent Gout Flares. Arthritis and Rheumatology 2019; 71: 1580-1586. Article. DOI: 10.1002/art.40896. |
| 495 | Zhang Y, Pan R, Xu Y, et al. Treatment of refractory gout with TNF-α antagonist etanercept combined with febuxostat. Annals of palliative medicine 2020; 9: 4332-4338. Article. DOI: 10.21037/apm-20-2072. |
| 496 | Zhenbin L and Ma X. Influnce of total glucosides of paeon on IL-6, IL-1β, TNFα of rats with acute gouty arthritis. International Journal of Rheumatic Diseases 2016; 19: 111. Conference Abstract. DOI: 10.1111/1756-185X.12962. |
| 497 | Zhou M, Hua L, Wang YF, et al. Oral Huzhang granules for the treatment of acute gouty arthritis: protocol for a double-blind, randomized, controlled trial. Trials 2022; 23. Article. DOI: 10.1186/s13063-022-06188-x. |
| 498 | Zitko T. Calcium PyroPhosphate Deposition disease. Ceska Revmatologie 2021; 29: 150-160. Note. |
| 499 | Zuber M, Kubale R, Bonkhoff H, et al. Tophaceous gout of the wrist involving soft tissue and bone - A rare cause of a carpal tunnel syndrome. Aktuelle Rheumatologie 1996; 21: 40-43. Article. |
| 500 | Atorvastatin vs Colchicine in Decrease of Troponin I of High Sensitivity in Patients With Rheumatoid Arthritis. https://clinicaltrials.gov/study/NCT04056039 |
| 501 | The Effect of ColcHicine on the Incidence of Knee or Hip Replacements https://clinicaltrials.gov/study/NCT06578182 |
| 502 | Colchicine for Symptom and Inflammation in Knee Osteoarthritis https://clinicaltrials.gov/study/NCT02176460 |
| 503 | To Evaluate Drug Interactions Between XNW3009, Febuxostat, and Colchicine in Patients With Gout https://clinicaltrials.gov/study/NCT05324423 |
| 504 | Efficacy and Safety of Colchicine for the Prevention of Gout Flares During the Initiation of Allopurinol https://clinicaltrials.gov/study/NCT01451645 |
| 505 | Relation Between Blood Concentration of Colchicine and Response to Colchicine Treatment in Gout Flare https://clinicaltrials.gov/study/NCT03933007 |
| 506 | Colchicine as Treatment for People With Hand Osteoarthritis https://clinicaltrials.gov/study/NCT04601883 |
| 507 | Trial of Colchicine Versus Prednisone for the Treatment of Acute CPPD Arthritis https://clinicaltrials.gov/study/NCT03128905 |
| 508 | Colchicine for the Treatment of Osteoarthritis of the Knee https://clinicaltrials.gov/study/NCT03913442 |
| 509 | Bucillamine for the Treatment of Acute Gout Flare in Subjects With Moderate to Severe Gout https://clinicaltrials.gov/study/NCT02330796 |
| 510 | Safety and Efficacy of Genakumab for Injection in Patients With Gout Flare https://clinicaltrials.gov/study/NCT05936268 |
| 511 | The Drug-Drug Interaction of SHR4640, Febuxostat and Colchicine in Patients With Gout https://clinicaltrials.gov/study/NCT03131583 |
| 512 | Immune Molecular and Inflammatory Cytokines Dysfunction Analysis in Gout Patients With Different Urate Levels https://clinicaltrials.gov/study/NCT02060552 |
| 513 | Evaluation of Colchicine and Nonsteroidal Anti-inflammatory Drug Combination Therapy and Renal Function in Gout Patients https://clinicaltrials.gov/study/NCT05006001 |
| 514 | Colchicine Or Naproxen Treatment for ACute gouT https://clinicaltrials.gov/study/NCT01994226 |
| 515 | Open Label Safety/Efficacy Study of Arhalofenate in Combination With Febuxostat for Hyperuricemia in Gout Patients https://clinicaltrials.gov/study/NCT01416402 |
| 516 | A Study of SSGJ-613 in Gout Subjects Initiating Urate-Lowering Treatment. https://clinicaltrials.gov/study/NCT06270225 |
| 517 | A Study of the Efficacy of Canakinumab in Prevention of Acute Flares in Chronic Gout Patients Initiating Allopurinol Therapy (Core Study) and a Long-term Study of the Efficacy and Safety of Canakinumab in Patients With Gout (Extension Study) https://clinicaltrials.gov/study/NCT00819585 |
| 518 | Prednisolone Versus Colchicine for Acute Gout in Primary Care https://clinicaltrials.gov/study/NCT05698680 |
| 519 | MPC-004 for the Treatment of an Acute Gout Flare https://clinicaltrials.gov/study/NCT00506883 |
| 520 | Safety/Efficacy Study to Evaluate of MBX-102 in Combination With Allopurinol in Gout Patients https://clinicaltrials.gov/study/NCT01399008 |
| 521 | Dose Finding Study to Evaluate Safety and Efficacy of 3 Dosages of SAP 001. https://clinicaltrials.gov/study/NCT05690204 |
| 522 | Safety & Efficacy of Canakinumab (ACZ885) in Patients With Frequent Flares for Whom Nonsterodial Anti-Inflammatory Drug (NSAIDs) and/ or Colchicine Are Contraindicated, Not Tolerated or Ineffective https://clinicaltrials.gov/study/NCT01362608 |
| 523 | Topical Application to Relieve Gout: Efficacy Trial & Safety (TARGETS).https://clinicaltrials.gov/study/NCT04130204 |
| 524 | Low-dose Colchicine With or Without Stepwise Dose Titration of Febuxostat for Flare Prophylaxis in Gout https://clinicaltrials.gov/study/NCT04697602 |
| 525 | Safety and Efficacy Study of MBX-102 in Treatment of Hyperuricemia in Patients With Gout https://clinicaltrials.gov/study/NCT01336686 |
| 526 | Evaluate the PK, PD, and Safety of Arhalofenate in Combination With Febuxostat for Hyperuricemia in Patients With Gout https://clinicaltrials.gov/study/NCT02252835 |
| 527 | A Phase 4 Safety and Efficacy Study to Evaluate Lesinurad 200 mg in Participants With Gout and Renal Impairment https://clinicaltrials.gov/study/NCT03226899 |
| 528 | 尾-RELIEVED - REsponse in Acute fLare and In prEVEntion of episoDes of Re-flare in Gout - Extension 3 (E3) https://clinicaltrials.gov/study/NCT01470989 |
| 529 | Treat-to-Target Serum Urate Versus Treat-to-Avoid Symptoms in Gout https://clinicaltrials.gov/study/NCT04875702 |
| 530 | Efficacy and Safety of Extended Release and Immediate Release Febuxostat in Participants With Gout https://clinicaltrials.gov/study/NCT02139046 |
| 531 | Efficacy and Safety of Extended Release and Immediate Release Febuxostat in Participants With Gout and Moderate Renal Impairment https://clinicaltrials.gov/study/NCT02128490 |
| 532 | Safety and Efficacy of Genakumab for Injection in Patients With Gout Initiating Urico-lowering Therapy https://clinicaltrials.gov/study/NCT05936281 |
| 533 | Intensive Urate Lowering Therapy of Febuxostat Compared to Allopurinol on Cardiovascular Risk in Patients With Gout https://clinicaltrials.gov/study/NCT02500641 |
| 534 | Evaluate the Efficacy and Safety of Arhalofenate for Preventing Flares and Reducing Serum Uric Acid in Gout Patients https://clinicaltrials.gov/study/NCT02063997 |
| 535 | Initiation of Allopurinol at First Medical Contact for Acute Attacks of Gout https://clinicaltrials.gov/study/NCT01310673 |
| 536 | Canakinumab in the Treatment of Acute Gout Flares and Prevention of New Flares in Patients Unable to Use Non-steroidal Anti-inflammatory Drugs (NSAIDs) and/or Colchicines Including a 12 Week Extension and a 1 Year Open-label Extension Study. https://clinicaltrials.gov/study/NCT01080131 |
| 537 | Targeted Dose Finding of Canakinumab (ACZ885) for Management of Acute Flare in Refractory or Contraindicated Gout Patients https://clinicaltrials.gov/study/NCT00798369 |
| 538 | Canakinumab in the Treatment of Acute Gout Flares and Prevention of New Flares in Patients Unable to Use Non-steroidal Anti-inflammatory Drugs (NSAIDs) and/or Colchicine Including a 12 Weeks Extension and an Open-label 48 Weeks Extension Study https://clinicaltrials.gov/study/NCT01029652 |
| 539 | Study of KRYSTEXXA庐 (Pegloticase) Plus Methotrexate in Participants With Uncontrolled Gout https://clinicaltrials.gov/study/NCT03994731 |
| 540 | Study of Pegloticase (KRYSTEXXA庐) Plus Methotrexate in Patients With Uncontrolled Gout https://clinicaltrials.gov/study/NCT03635957 |
| 541 | Levent Ediz.elt Effect of colchicine on total antioxidant capacity, antioxidant enzymes and oxidative stress markers in patients with knee osteoarthritis. Int J Clin Med. 2012;3(05):377 |
| 542 | Amirpour A, Mousavi M, Abolghasemi R, Taziki O, VH K. The Effect of Colchicine in Improving the Symptoms of Patients with Knee Osteoarthritis. J Babol Univ Med Sci. 2016;18(11):7-13. PubMed PMID: 36143334; PubMed Central PMCID: PMCPMC9500941. |
| 543 | Erden M, Ediz L, Tuluce Y, Ozkol H, Toprak M, F D. Effect of Colchicine on Total Antioxidant Capacity, Antioxidant Enzymes and Oxidative Stress Markers in Patients with Knee Osteoarthritis. International journal of rheumatic diseases. 2012;3(05):377. Epub 2017/03/07. doi: 10.1111/1756-185x.13022. PubMed PMID: 28261974. |
