# Supplementary material for: Colchicine combination therapy increases treatment tolerance in patients with arthritis: A systematic review and meta-analysis
Source: PLoS One. 2024 Dec 30;19(12):e0316126. doi: 10.1371/journal.pone.0316126 (PMC11684588; doi:10.1371/journal.pone.0316126)
Supplement: Attachment — Submitted filename: Response to Reviewers.docx [file pone.0316126.s002.docx]

Dear editors and reviewers：

Thank you to the editors and reviewers for help with my paper and for providing suggestions to help me improve my paper, I would appreciate your time and effort！

Here's what I've done to this paper！

***For Journal Requirements:***

* Please ensure that your manuscript meets PLOS ONE's style requirements, including those for file naming. The PLOS ONE style templates can be found at https://journals.plos.org/plosone/s/file?id=wjVg/PLOSOne_formatting_sample_main_body.pdf and <https://journals.plos.org/plosone/s/file?id=ba62/PLOSOne_formatting_sample_title_authors_affiliations.pdf>

-------Thanks for the reminder, the revised manuscript meets PLOS ONE's style requirements exactly

* We noticed you have some minor occurrence of overlapping text with the following previous publication(s), which needs to be addressed:https://doi.org/10.3389/fnagi.2022.985109

------Thank you for your opinion, we read the article（Risk of dementia or cognitive impairment in non-alcoholic fatty liver disease: A systematic review and meta-analysis），This is also a meta-analysis article, but it is different from our topic, and we may have overlapped in the method steps, all of which are in databases such as PubMed, Excerpta Medica Database (EMBASE), Cochrane Library, etc. And the method used is the same. Therefore, we have further refined the method steps to reduce overlap.

* Thank you for stating the following in your Competing Interests section:

"The authors declare that they have no competing interests"

Please complete your Competing Interests on the online submission form to state any Competing Interests. If you have no competing interests, please state "The authors have declared that no competing interests exist.", as detailed online in our guide for authors at http://journals.plos.org/plosone/s/submit-now

This information should be included in your cover letter; we will change the online submission form on your behalf.

-----Thanks for the reminders and comments, we have added to the cover letter, the last sentence of the first paragraph.

Thank you for helping me resubmit, and our supplementary materials have also been modified, and I hope to re-upload the supplementary materials, thank you for your help.

* PLOS requires an ORCID iD for the corresponding author in Editorial Manager on papers submitted after December 6th, 2016. Please ensure that you have an ORCID iD and that it is validated in Editorial Manager. To do this, go to ‘Update my Information’ (in the upper left-hand corner of the main menu), and click on the Fetch/Validate link next to the ORCID field. This will take you to the ORCID site and allow you to create a new iD or authenticate a pre-existing iD in Editorial Manager. Please see the following video for instructions on linking an ORCID iD to your Editorial Manager account: [https://www.youtube.com/watch?v=_xcclfuvtxQ](https://www.youtube.com/watch?v=_xcclfuvtxQ---------)

There is also corresponding ORCID iD on the first page of the article

Changwei Zhao：<https://orcid.org/0000-0001-7211-2798>

Wenhai Zhao：<https://orcid.org/0009-0001-8189-2148>

Xiangxin Li：https://orcid.org/0009-0009-9297-8180

* Please include captions for your Supporting Information files at the end of your manuscript, and update any in-text citations to match accordingly. Please see our Supporting Information guidelines for more information: http://journals.plos.org/plosone/s/supporting-information.

--------Thanks to this reminder, it was very helpful for us, and we also revised the corresponding references when we revised the manuscript.

***For Reviewer #1 ：***

Thank you for your review of this post and the advantages, disadvantages, and suggestions raised. We did our best to make changes based on your comments，and here are the changes I made and replied to.

* Small Number of Studies: Only eight studies were included, which limits the statistical power and the generalizability of the findings. The small sample size of included studies can lead to less reliable results and a higher margin of error.

------Thanks to the reviewers' comments, we re-screened the literature in the database; thank you for your suggestion; we re-screened the relevant literature in the database and expanded it to 2024.9.4, according to the inclusion criteria and exclusion criteria. We added three articles, which are about calcium pyrophosphate crystal arthritis, psoriatic arthritis and gouty arthritis, and we will continue to pay attention to the database in the future to improve our research

*	Over-reliance on Patient-reported Outcomes: The significant improvement in the patient’s global assessment score may be influenced by subjective perceptions rather than objective measures of improvement. More emphasis should be placed on objective clinical outcomes.

-------Thanks to the comments of the reviewers, most of the studies we included used overall ratings, such as pain on patients as a finding rather than some objective measure, so we were unable to combine them. However, based on your opinion, we have also described these indicators, as described in 3.7 Biomarkers and imaging markers. The impact on Biomarkers and imaging markers has also been added to the the second paragraph of 4 discussion and is marked in red.

*	High Heterogeneity: Significant heterogeneity (I² > 90%) in some outcomes indicates that the studies are not sufficiently comparable. This could be due to differences in study design, populations, dosages, and outcome measurements. High heterogeneity limits the reliability of pooled estimates and makes it difficult to draw firm conclusions.

------Thank you! We examined the design differences of the included studies in detail and provided the specific design of each study in the table to better understand the impact of these differences on the results. In terms of analysis methods, Cohen's d method was also introduced to facilitate standardized data processing and reduce bias caused by measurement methods. Due to the lack of relevant studies, our sensitivity analysis was not sufficient to reduce the heterogeneity of the results, but we believe that the significance of the results is still valuable for reference.

*	Risk of Bias: Two studies (23 and 24) had a high risk of bias due to unclear randomization methods, and did not use blinding. This compromises the internal validity of the results.

-------Thanks to the comments of the reviewers, we revised the search strategy for the articles, expanded the search dates, read the articles further, emailed the authors for uncertainty and found that all studies were blinded. However, three studies did not describe the method of randomisation, these factors indeed put these studies at a higher risk of bias. The risk of bias could potentially impact the variation in relative risk scores. Studies with inadequate randomization and blinding are more susceptible to systematic errors, which can lead to overestimation or underestimation of the treatment effects. This variability can contribute to the observed heterogeneity in the RR scores. We added a risk of bias figure and described it in the article.

*	Variable Control Treatments: The control treatments were not standardized, which introduces variability and makes it challenging to attribute differences in outcomes solely to the colchicine treatment.

-------Thanks to the comments of the reviewers, we have added specific measures of conventional treatment to the article(2.3 Inclusion criteria 2 intervention:Conventional treatment includes topical analgesics, supplements, non-steroidal anti-inflammatory drugs and physiotherapy.

*	Limited Scope of Arthritis Types: The study primarily focuses on gouty arthritis (GA) and osteoarthritis (OA) and does not separately analyze other types like rheumatoid arthritis and juvenile idiopathic arthritis. This limits the applicability of the findings to other forms of arthritis.

-----Thanks to the comments of the reviewers, we aimed to investigate the efficacy and safety of colchicine in arthritis, and in order to increase the number of studies, we expanded the search strategy to 4 September 2024, and enriched the results by including calcium pyrophosphate crystal arthritis and psoriatic arthritis according to the inclusion and exclusion criteria. We will continue to monitor the database in the future, and if there are new studies such as rheumatoid arthritis and juvenile idiopathic arthritis, we will continue to include them to improve limitations.

*	Inconsistent Outcome Reporting: The study reports mixed results with some outcomes showing significant improvement (e.g., patient’s global assessment) while others do not (e.g., VAS score, WOMAC scores). This inconsistency raises questions about the overall efficacy of colchicine.

------Our studies were based on the analysis of the included literature, and the results of most of the included RCTs were mainly subjective. Colchicine can benefit patients' global assessment, etc., but does not improve significantly on other outcomes. This requires larger, more clinical studies to validate the efficacy and safety of colchicine for arthritis.

*	Dosage Variability: Differences in colchicine dosages across studies could affect the outcomes. The study should standardize dosages or analyze the impact of dosage variations.

------Thank you for your suggestion, we have added the dose to Table 1, and we will continue to monitor the changes in the database to further analyze the efficacy and safety analysis of colchicine dosage for arthritis

*	Short Follow-up Periods: Some studies included in the analysis had relatively short follow-up periods. Long-term effects and safety of colchicine in combination therapy need more extended evaluation.

-------Thanks to the reviewers' comments, we did not perform pooled analyses as we did not include a measure for long-term efficacy in the included literature. In response to your suggestion, we have reviewed the relevant literature to supplement it in the discussion section, as detailed in: Seventh paragraph of the 4 discussion

* Increase Sample Size and Number of Studies: Conduct more RCTs with larger sample sizes to improve the robustness and generalizability of the findings.

-------We reinstated the search strategy and extended the date to 4 September 2024 to include three studies on calcium pyrophosphate crystal arthritis, psoriatic arthritis, and gouty arthritis, according to the inclusion criteria, and we modified the search process, search results, and results in the articles. In the future, we will continue to monitor the database to improve our research.

* Standardize Control Treatments: Use more consistent control treatments across studies to reduce variability and improve comparability.

--------Thanks to the comments of the reviewers, we have added specific measures of conventional treatment to the article(2.3 Inclusion criteria 2 intervention:Conventional treatment includes topical analgesics, supplements, non-steroidal anti-inflammatory drugs and physiotherapy.In the future, we will continue to monitor the updating of the literature，to standardize Control Treatments

* Focus on Objective Outcomes: Incorporate more objective measures of improvement, such as imaging studies or biomarkers, to complement patient-reported outcomes.

------In the included literature, there were insufficient numbers of indicators (imaging studies or biomarkers) that could not be combined, so we did not use them to analyze them. Based on your suggestion, we have supplemented and discussed these indicators in 3.7 Biomarkers and imaging markers,and it is also discussed in the second paragraph of 4 discussion.

* Long-term Safety Assessment: Evaluate the long-term safety and efficacy of combined colchicine therapy to better understand its risk-benefit profile.

.-----Thanks for the suggestion. We did not perform pooled analyses as long-term efficacy measures were not included in our included literature. In response to your suggestion, we have reviewed the relevant literature and supplemented it in the discussion section, as detailed in paragraph 7 of the 4 discussion section

* Expand Scope to Other Types of Arthritis: Include and separately analyze other forms of arthritis, such as rheumatoid arthritis and juvenile idiopathic arthritis, to broaden the applicability of the findings.

-----Thank you for your suggestion; we expanded the search strategy to 4 September 2024 and re-screened the relevant articles in the database; according to the inclusion and exclusion criteria, we added three articles on calcium pyrophosphate crystal arthritis, psoriatic arthritis and gouty arthritis, and we will continue to monitor the database in the future to improve our studies.

***For Reviewer #2 ：***

Thank you for your praise of this paper and for your affirmation of this topic. We did our best to make changes based on your comments，and here are the changes I made and replied to.

*	In figure 3 (WOMAC pain score), figure 4 (WOMAC function score) and figure 5 (Total WOMAC scale score), the I2 value for heterogeneity was found to be 0, which indicates homogeneity. Which model was used to assess heterogeneity for these figures? Also, can the authors comment on sampling error issues?

------Model Used to Assess Heterogeneity: For figures 3 (WOMAC pain score), 4 (WOMAC function score), and 5 (Total WOMAC scale score), we used the random-effects model to assess heterogeneity. The I² value of 0 indicates homogeneity among the included studies, suggesting that the observed variability is likely due to sampling error. Sampling Error Issues: While the I² value suggests homogeneity, it is important to consider potential sampling errors. Sampling error can arise from various sources, including small sample sizes, variations in study populations, and differences in measurement techniques. We acknowledge that these factors could influence the results, and we have taken steps to minimize their impact by standardizing data processing methods, such as using Cohen’s d for effect size calculations. Despite these efforts, some degree of sampling error is inevitable, and we recommend interpreting the results with this consideration in mind.

• The risk of adverse events showed an RR score of 1.30 with statistically no significant difference between groups. Authors could benefit from discussing two things here:

*	1.The weight percentages assigned to studies seem very different for adverse event assessment. Because these studies were included in the adverse event assessment and two of the studies (Ref. 18 and 22) show a higher RR for adverse events with much lower weight percentages 7.68% and 0.73% respectively, could the authors explain the reason for choosing these weight percentages?

-----The weight assigned to each study in the meta-analysis depends on the sample size and the variability of the study results. Larger studies with less variability typically receive higher weights because their results are considered more reliable. Smaller studies or those with higher variability might receive lower weights, even if they show higher relative risks. This is to prevent less reliable studies from disproportionately influencing the overall results.

*	2.Authors state that 4 out of 8 of the selected studies did not describe a specific method of randomization. Also, two of the selected studies did not mention blinding (one of which is part of the adverse assessment), putting them at a higher risk of bias. Could the risk of bias have some impact on the variation in RR scores? It may be pertinent to add some explanation in the manuscript based on the analysis.

----We corrected the randomization methods and blinding methods by further reading the literature, reviewing the literature, and contacting the authors, and explaining them in the article. However, there were still 3 studies that did not mention the randomization method,these factors indeed put these studies at a higher risk of bias. The risk of bias could potentially impact the variation in relative risk scores. Studies with inadequate randomization and blinding are more susceptible to systematic errors, which can lead to overestimation or underestimation of the treatment effects. This variability can contribute to the observed heterogeneity in the RR scores. We will add a section in the manuscript to discuss the potential impact of these biases on our results.

*	Ref. 20 was only used to assess the risk from adverse events (Ref. 20 focuses on acute GOA) and not included in any other assessment. Upon looking into Ref. 20, the data were not available for any other assessment in the RCT. Do the authors think this RCT is valid for this manuscript? Also, was it the author’s intention to do the meta-analysis on HOA and KOA? In that case, do the authors believe that the manuscript would benefit from refocusing on these two, rather than the overarching topic of Arthritis?

-------Thanks for the suggestion, although in this article VAS score is the primary outcome and WOMAC pain score is a secondary outcome, adverse effects are also one of our outcomes, and we found that colchicine does not increase adverse effects, so we included this review.

Thanks to the reviewers for their suggestion to focus on HOA and KOA, but there is currently relevant literature analyzing osteoarthritis, so we chose arthritis as our topic, and further sub-explanations are made in the article, and they are marked in red(The first paragraph of 4 discussion).

We also expanded the search to 4 September 2024 to include several studies, including calcium pyrophosphate crystal arthritis and psoriatic arthritis, but there were too few studies to be combined, so we discussed them separately and added results in the biochemical section.

*	Continuing from the last point, Ref 25 in the manuscript did a systematic review on efficacy and safety of Colchicine on HOA and KOA. Can the authors comment on how the manuscript may be different from the already published work? There are quite a few overlaps between the current manuscript and Ref. 25, including the selection of RCTs.

------Previous literature has evaluated the efficacy and safety of colchicine in the treatment of osteoarthritis.Colchicine is an alkaloid It possesses anti-inflammatory, anti-fibrotic, and cardiovascular protective effects. In addition to osteoarthritis, colchicine is also used to treat gouty arthritis, psoriatic arthritis, etc., so this paper is aimed at the relationship between arthritis and colchicine, and our purpose is to evaluate the correlation between colchicine and arthritis, including osteoarthritis, rheumatoid arthritis, gouty arthritis, etc., but in the end we only included osteoarthritis and gouty arthritis, calcium pyrophosphate crystal arthritis, psoriatic arthritisso we are trying to illustrate the efficacy and safety of colchicine in the treatment of arthritis. we added it in the article and it is marked in red(The first paragraph of 4 discussion).

*	The manuscript could benefit from risk of bias analysis as a figure representation to highlight the parameters of bias analysis in selected RCTs.

-----Thank you for your opinion！Based on your suggestion, we have supplemented the figure of risk of bias analysis（Figure2）

*	What is considered conventional treatment (CT)? Was it the same in different RCTs? The manuscript could benefit from summarizing what the variety of conventional treatment used were in the selected RCTs.

----------Thank you for the help of this article, we think that the conventional treatment is the common treatments for arthritis mentioned in the article, the routine treatment in each paper is different, by carefully reading the literature again, we have further modified and improved the table, and the article has been supplemented and marked in red (2.3 Inclusion criteria), Conventional treatment includes topical analgesics, supplements, non-steroidal anti-inflammatory drugs and physiotherapy, and the table has also been modified in detail.

*	The authors could benefit from adding information regarding dosing of Colchicine in combination therapy. I recognize that the authors mentioned most studies selected used 0.5 mg twice daily as the dosage, but each RCT associated dosage should be mentioned in Table 1.

-------Thanks to this comment, we have added colchicine dosage supplementation to Table 1 and it is marked in red. Most studies were 0.5 mg twice daily or 1.5 g daily, or the dose could be adjusted while taking the drug.

*	Minor language corrections:

• In methods, Section 2.2, first line, the word “library” is misspelled.

• In methods, Section 2.2, Line 5, the word “Colchicine” is mentioned twice.

• In results, Section 3.4.1, Line 8 “VAS” is mislabeled as “VSA”

• In discussion section, Page 25 of the manuscript, the following line is aberrant:

“…and its binding to microtubulin and disruption of microtubule network could result in impaired protein assembly, decreased cytosolization and cytosolization, altered cellular morphology, cellular The binding…”

-----Thank you for your careful comments on this article, we have made changes and annotations in accordance with your comments, and the fourth point is to describe the mechanism of colchicine adverse reactions, according to your comments we have made changes and additions, and revised the references
