# Supplementary material for: Colchicine combination therapy increases treatment tolerance in patients with arthritis: A systematic review and meta-analysis
Source: PLoS One. 2024 Dec 30;19(12):e0316126. doi: 10.1371/journal.pone.0316126 (PMC11684588; doi:10.1371/journal.pone.0316126)
Supplement: Attachment — Submitted filename: Response to Reviewers.docx [file pone.0316126.s003.docx]

Dear editors and reviewers：

Thank you to the editors and reviewers for help with my paper and for providing suggestions to help me improve my paper and giving me the opportunity to revise it. I would appreciate your time and effort！

Here's what I've done to this paper！

PONE-D-24-18974R1

Colchicine combination therapy increases treatment tolerance in patients with arthritis: a systematic review and meta-analysis

1. The study focusing on Calcium pyrophosphate crystal arthritis (Pascart et.al) has not been mentioned in any of the analysis or the newly added “Biomarkers and Imaging markers” section. I’m not sure how this study is relevant or how it supports any conclusions. I suggest providing some analysis on this study in the main text.

Thank you for your valuable comments, our inclusion included all arthritis as well as calcium pyrophosphate crystal arthritis, so we included this article (Pascart et.al). Based on your suggestion, we carefully read the literature again and added a description of the effect of colchicine ＋ CT versus prednisone ＋ CT in the treatment of calcium pyrophosphate crystal arthritis in Line376-379 and marked in red.

2. Also, Pascart et.al conducted the study for 24 hours, as compared to several weeks/months in all other selected RCTs. How do the authors justify the selection of this study from a “Duration of treatment” perspective?

Thank you for your valuable comments on manuscript, the primary outcome measure given in the paper (Pascart et.al) is the day 2 (24 hours), mainly to observe the short-term efficacy of CT + colchicine and CT + prednisone in the treatment of acute calcium pyrophosphate crystal arthritis, and the primary outcome measure is change from baseline pain. measured by the VAS from (0 to 100 mm) recorded on day 2 (24 h) for the most painful. Due to the short duration of treatment compared to other RCTs, we did not combine the analyses but selected for inclusion. We describe the results in Line 376-379 and add limitations (L418-421), which are marked in red.

3. The final addition of Mckendry et.al for Psoriatic Arthritis does not have any analysis provided, except for the statement “McKendry, R found that psoriatic arthritis laboratory measures were unchanged during colchicine treatment”. How do the laboratory measures provide justification for advantages of combination therapy? This statement needs to be expanded to provide analysis for evidence on how it supports the central message of Colchicine + CT being better than CT itself.

Thank you for your comments. According to the inclusion and exclusion criteria, this literature on the treatment of psoriatic arthritis by Mckendry et.al needs to be included, although he has no indicators that can be pooled, and there are no data to prove the advantage of combination therapy. However, in order to make the data more realistic and objective, we did not exclude this article and supplemented the discussion of this article in the discussion section (Line364-369).

4. In the adverse events analysis, the weight percentages have been significantly changed from the first version of the manuscript, with only the addition of one additional study. While I accept the authors claim that larger studies with less variability will be assigned a higher weight, it seems highly irregular that the weights would be so different from the initial values chosen in the first version of the manuscript by the authors, as compared to now (SK DAS 2002b went from 36.89% to 1.45%). Could the authors kindly explain the reason for this change?

Thank you, in the first revision, we expanded the literature screening time and reorganized all the data, and the doubts were discussed and adjudicated by a third person. We considered the problem of pooled analysis of 0 values in some adverse reactions, so the default estimate was 1. Later, I consulted more papers, and when I reorganized and re-analyzed, I found that the value of 0 did not affect the results, so I kept the value of 0. So the weights have changed. However, this does not affect the judgment of the result. At the same time, we have checked other data to ensure its accuracy.

I appreciate the author’s addition of Risk of Bias as a figure representation (Figure 2) and updating Table 1 to reflect the requested information. My concerns are associated with the fact that all chosen studies should provide evidence towards the central conclusion “In combination with colchicine, the quality of life of patients was improved without increasing the incidence of adverse events”. I firmly believe that by providing some further analysis, the manuscript can be improved significantly.

Thank you for your acknowledgment of my revisions, your comments are very useful for the manuscript, and I will continue to improve it so that the paper can be significantly improved.

*	Minor Corrections:

Line 287: “Colchicine” and Tricyclic” is misspelled.

Thank you for your careful comments on this article, we have made changes and annotations in accordance with your comments, according to your comments we have made changes and additions, and revised the references.
